# Bifunctionality of Re Supported on TiO_2_ in Driving Methanol Formation in Low-Temperature CO_2_ Hydrogenation

**DOI:** 10.1021/acscatal.3c01599

**Published:** 2023-08-01

**Authors:** Nat Phongprueksathat, Kah Wei Ting, Shinya Mine, Yuan Jing, Ryo Toyoshima, Hiroshi Kondoh, Ken-ichi Shimizu, Takashi Toyao, Atsushi Urakawa

**Affiliations:** † Catalysis Engineering, Department of Chemical Engineering, Delft University of Technology, Van der Maasweg 9, Delft 2629 HZ, Netherlands; ‡ Institute for Catalysis, Hokkaido University, N-21, W-10, Sapporo 001-0021, Japan; § Department of Chemistry, Keio University, 3-14-1 Hiyoshi, Kohoku-ku, Yokohama 223-8522, Japan

**Keywords:** CO_2_ hydrogenation, methanol, rhenium, TiO_2_, reaction mechanism

## Abstract

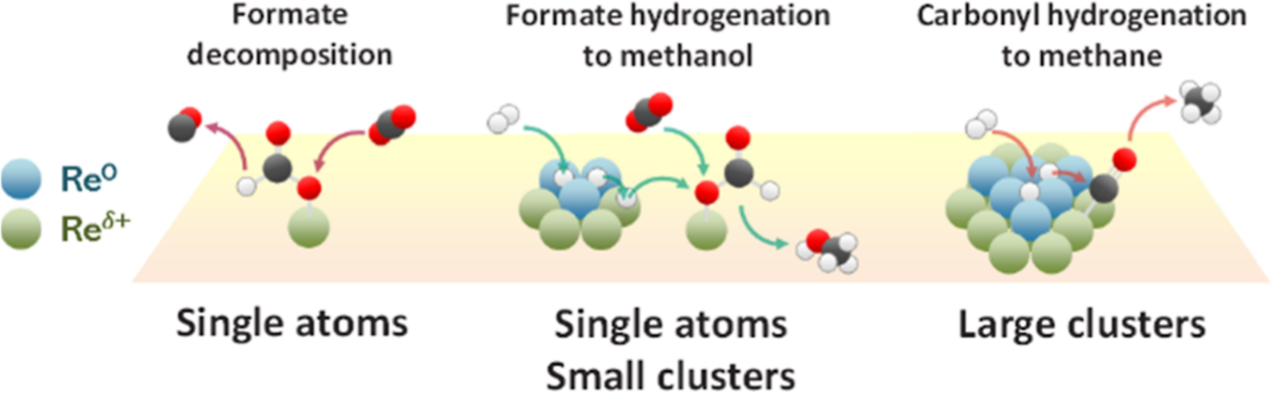

Low temperature and high pressure are thermodynamically more favorable conditions to achieve high conversion and high methanol selectivity in CO_2_ hydrogenation. However, low-temperature activity is generally very poor due to the sluggish kinetics, and thus, designing highly selective catalysts active below 200 °C is a great challenge in CO_2_-to-methanol conversion. Recently, Re/TiO_2_ has been reported as a promising catalyst. We show that Re/TiO_2_ is indeed more active in continuous and high-pressure (56 and 331 bar) operations at 125–200 °C compared to an industrial Cu/ZnO/Al_2_O_3_ catalyst, which suffers from the formation of methyl formate and its decomposition to carbon monoxide. At lower temperatures, precise understanding and control over the active surface intermediates are crucial to boosting conversion kinetics. This work aims at elucidating the nature of active sites and active species by means of *in situ/operando* X-ray absorption spectroscopy, Raman spectroscopy, ambient-pressure X-ray photoelectron spectroscopy (AP-XPS), and diffuse reflectance infrared Fourier transform spectroscopy (DRIFTS). Transient *operando* DRIFTS studies uncover the activation of CO_2_ to form active formate intermediates leading to methanol formation and also active rhenium carbonyl intermediates leading to methane over cationic Re single atoms characterized by rhenium tricarbonyl complexes. The transient techniques enable us to differentiate the active species from the spectator one on TiO_2_ support, such as less reactive formate originating from spillover and methoxy from methanol adsorption. The AP-XPS supports the fact that metallic Re species act as H_2_ activators, leading to H-spillover and importantly to hydrogenation of the active formate intermediate present over cationic Re species. The origin of the unique reactivity of Re/TiO_2_ was suggested as the coexistence of cationic highly dispersed Re including single atoms, driving the formation of monodentate formate, and metallic Re clusters in the vicinity, activating the hydrogenation of the formate to methanol.

## 1 Introduction

Recycling fossil fuel-derived carbon dioxide (CO_2_) by converting it into chemicals or fuels such as methanol, ethanol, and dimethyl ether is a promising approach toward alleviating the impact of global warming.^1^ Among those chemicals, methanol is one of the most versatile chemicals as an energy carrier and an alternative petrochemical feedstock toward a less fossil-fuel-dependent and/or circular economy, known as the “methanol economy”.^2^

Methanol can be synthesized on industrially relevant scales via CO_2_ hydrogenation over the most known Cu/ZnO/Al_2_O_3_ catalysts at 220–250 °C and 20–100 bar.^3−5^ According to Le Châtelier’s principle, however, lower temperature and higher pressure than the aforementioned conditions are more thermodynamically favorable. Taking both chemical and vapor–liquid equilibria into the thermodynamic calculation, conditions below 200 °C and above 150 bar are required to achieve nearly full CO_2_ conversion and CH_3_OH selectivity.^6^ This is thanks to the *in situ* separation of condensable products (e.g., methanol and water) from reactant gases, thereby driving forward the reaction equilibrium.^7,8^ Furthermore, operating the reaction below 200 °C is expected to be not only beneficial by reducing energy consumption but also alleviating deactivation due to sintering.

In practice, low-temperature high-pressure CO_2_ hydrogenation over a Cu/ZnO/Al_2_O_3_ catalyst yields high CO selectivity below 260 °C despite the thermodynamically favorable conditions toward methanol formation (*P* = 331 bar and H_2_/CO_2_ = 10).^9^ At and above 260 °C, the reaction mechanism of methanol formation appears as reverse water–gas shift and subsequent CO hydrogenation according to the residence time and space-resolved *operando* studies.^10^ CO hydrogenation was kinetically limited at a lower temperature, whereas the direct CO_2_ hydrogenation to methanol is the major path at a low temperature (180 °C). The reaction path may be condition-dependent (e.g., on pressure and catalyst); one study reports contrary results where the source of carbon can gradually shift from CO_2_ to CO as the temperature decreases toward 160 °C.^11^ In literature, it is widely accepted that using Cu-based catalysts CO_2_ is the main source of carbon in CH_3_OH and that CO is formed independently via different intermediates and converted to methanol via CO hydrogenation by an order of magnitude slower than CO_2_ hydrogenation.^12,13^ Moreover, CH_3_OH formed via direct CO_2_ hydrogenation can decompose to CO.^10^ Lewis-acidic sites over Al_2_O_3_ play a key role in CH_3_OH decomposition to CO via a methyl formate-mediated pathway.^14^ Thus, developing novel catalysts with well-defined active sites and a clear understanding of the reaction mechanisms are crucial for suppressing CO formation while achieving high CO_2_ conversion and CH_3_OH selectivity.

Supported rhenium (Re) catalysts show remarkable potential in heterogeneous catalysis, especially for CO_2_ hydrogenation.^15−18^ Recently, Re/TiO_2_ was found to exhibit the highest turnover frequency and CH_3_OH selectivity among various metal catalysts (Co, Ni, Cu, Ru, Rh, Pd, Ag, Re Ir, and Pt) supported on TiO_2_, bulk Re catalysts (Re^0^, NH_4_ReO_4_, ReO_2_, ReO_3_, and Re_2_O_7_), and Re supported on various supports (ZrO_2_, Al_2_O_3_, SiO_2_, C, CeO_2_, MgO, SiO_2_–Al_2_O_3_, SnO_2_, H-ZSM-5, and HY) under a batch reaction condition at 150 °C and 60 bar (H_2_/CO_2_ = 5, 24 h).^19^ Despite the uniquely high activity, the major challenge of the catalyst is the formation of CH_4_ as a byproduct. CH_4_ selectivity over Re/TiO_2_ is promoted not only by the reaction conditions (higher reaction temperature and longer contact time)^20,21^ but also by the catalyst structures such as larger Re cluster size and Re oxidation state.^19^ The structural chemistry of Re on metal oxide support materials is complex. The reduction of Re^7+^ (Re_2_O_7_) supported on γ-Al_2_O_3_ can disperse Re particles into majorly single Re atoms, Re nanoclusters, and unreduced Re (due to the oxophilic nature).^22^ After a similar reduction treatment to Re_2_O_7_/TiO_2_, subnanometer Re clusters are formed with oxidation numbers between Re^0^ and Re^4+^ that were suggested to be favorable for high CH_3_OH selectivity.^19^ The fluxional oxidation states of both Re and Ti can influence the nature of the acid site on Re/TiO_2_; the hydroxyl group attracted by Re^6+^ or Re^6+^ generates Brønsted acid sites, while the incorporation of Re^4+^ into TiO_2_ forming Re^*n*+^–O–Ti^4+^(Ti^3+^) bonds creates Lewis acid sites.^23^ For Cu-based catalysts, the Lewis acid strength of Ti sites next to Cu can stabilize surface intermediates such as formate and methoxy in proximity to metal nanoparticles leading to boosting the CH_3_OH formation rate.^24,25^ For Re/TiO_2_, *in situ* IR showed that formate and methoxy are possible intermediates for CH_3_OH formation, while carbonyls are a possible intermediate for CH_4_ formation.^19,20^ However, the observable surface species under the steady-state diffuse reflectance infrared Fourier transform spectroscopy (DRIFTS) experiment could be both active or spectator species. Elucidation of the precise reaction mechanisms and involved active species requires more advanced approaches such as transient techniques with periodic external stimulus for species discrimination.^26−28^ Transient techniques are applicable for pinpointing the state and structure of active sites from multi-oxidation state Re particles and understanding the interrelationship between the nature of active sites and surface intermediates.

In this study, the catalytic activity of Re/TiO_2_ was investigated at low temperature and high pressure of 150 °C and 331 bar. The results from various *in situ/operando* spectroscopy techniques such as DRIFTS, X-ray absorption spectroscopy (XAS), Raman spectroscopy, and ambient-pressure X-ray photoelectron spectroscopy (AP-XPS) provide insights into surface species, the oxidation state of Re, and the structural change of TiO_2_. DRIFTS combined with steady-state isotopic transient kinetic analysis (SSITKA) using D_2_ and ^13^CO_2_ elucidate distinct surface intermediates and pathways toward CH_3_OH and CH_4_ formation. Correlating the surface species, catalyst structures, and outlet gas product, we provide molecular-level insights into the reaction mechanisms and the nature of active sites, which could further assist the development of superior catalysts.

## 2 Results and Discussion

### 2.1 Selectivity Progression during Low-Temperature CO_2_ Hydrogenation at High Pressure

The catalytic performances of 3 wt % Re/TiO_2_ and commercial Cu/ZnO/Al_2_O_3_ (64/25/10 wt %) were studied at the stoichiometric H_2_/CO_2_ ratio of 3 at 125–200 °C and 56 bar (reactant pressure, the reaction pressure is 60 bar including an inert gas for calibration). As shown in Figure S1a, CO_2_ conversion over both catalysts increases with temperatures. However, the temperature of 150 °C was optimal for the highest CH_3_OH selectivity (Figure S1b). Increasing temperature promotes CO selectivity over both catalysts (Figure S1c) as well as CH_4_ selectivity (over Re/TiO_2_, Figure S1d) in contrast to HCOOCH_3_ selectivity (Figure S1e). Despite higher CH_3_OH selectivity, the CO_2_ conversion of Cu/ZnO/Al_2_O_3_ was limited at 150 °C (below 1%) in comparison to Re/TiO_2_ (ca. 4%), which highlights the significance of the latter catalyst in low-temperature operations.

**Figure 1:**
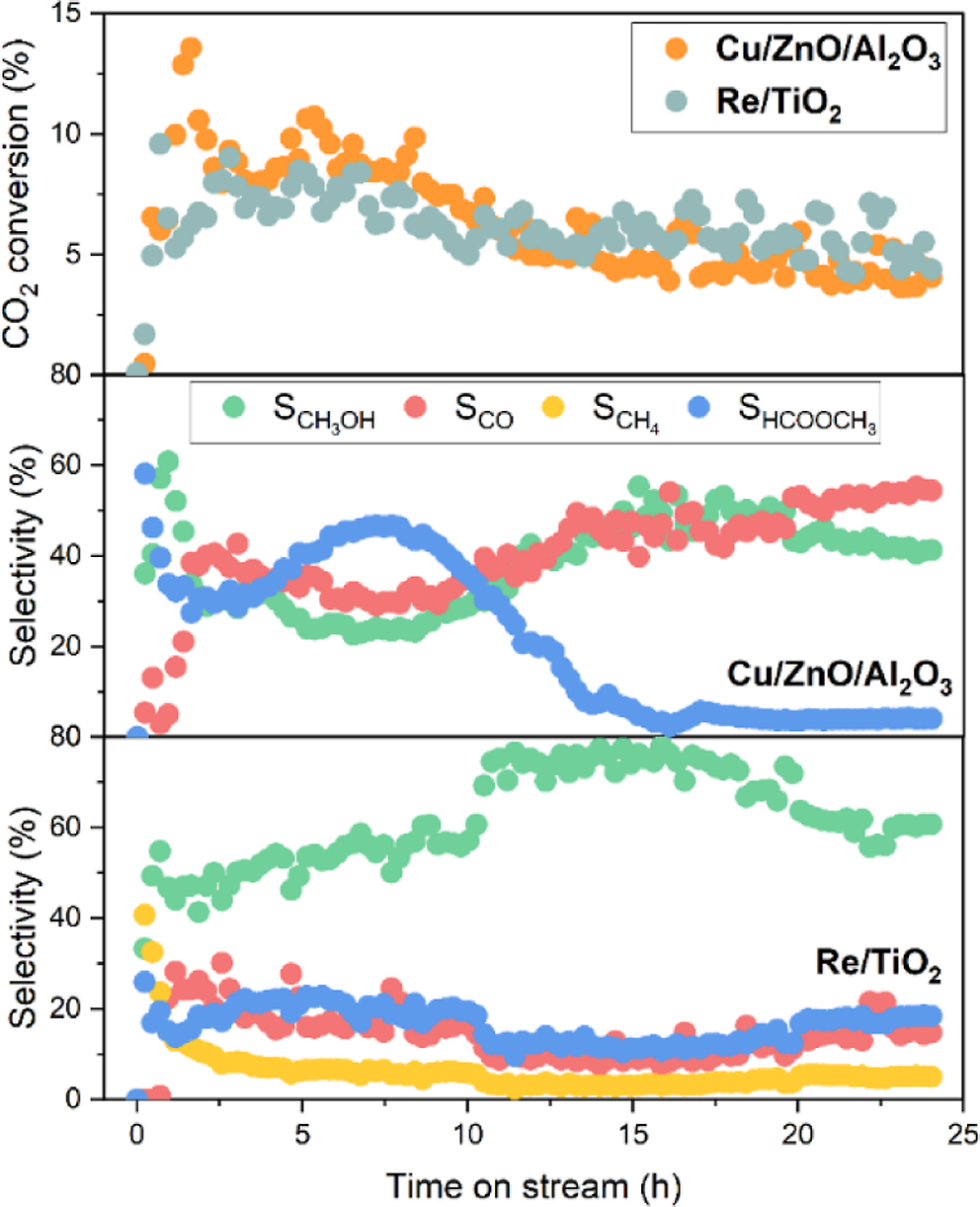
Catalytic activity of Cu/ZnO/Al_2_O_3_ and 3 wt % Re/TiO_2_ in CO_2_ hydrogenation. H_2_/CO_2_ = 3, *T* = 150 °C, *P* = 331 bar, and GHSV = 2000 h^–1^ (2 NL g_cat_^–1^ h^–1^).

At the stoichiometric H_2_/CO_2_ ratio of 3, increasing pressure did not only provide considerable advantages in boosting CO_2_ conversion and CH_3_OH selectivity over Cu/ZnO/Al_2_O_3_ at 260 °C (up to 70% and 90%, respectively)^29^ but was also more thermodynamically favorable for full CO_2_ conversion and CH_3_OH selectivity below 200 °C.^6^ The catalytic performances at 150 °C were further investigated at a high pressure of 331 bar [360 bar reaction pressure for 24 h (Figure 1)]. Initially, both catalysts showed higher CO_2_ conversion that declined with time-on-stream, more prominently for Cu/ZnO/Al_2_O_3_. Re/TiO_2_ showed a comparable and higher CO_2_ conversion to Cu/ZnO/Al_2_O_3_ over time despite significantly lower metal loading. CH_3_OH selectivity displayed three distinguished stages within the 24 h experiment: activation (0–10 h), stable performance (10–20 h), and stabilization and deactivation (>20 h). During the activation period, methyl formate (HCOOCH_3_) is the main product over Cu/ZnO/Al_2_O_3_. After initial >60% selectivity followed by a decrease, HCOOCH_3_ selectivity reaches ca. 47% at ca. 8 h before a rapid decline to less than 5%. Along with the decrease of HCOOCH_3_ selectivity, CH_3_OH and CO selectivities gradually increase and become stable. On the other hand, Re/TiO_2_ shows a high initial CH_4_ selectivity (>40%) that rapidly declines within 5 h. HCOOCH_3_ selectivity over Re/TiO_2_ is lower than that of Cu/ZnO/Al_2_O_3_ during the first 10 h, but it becomes higher over time. Generally, the drops in HCOOCH_3_ selectivity accompany a counteracting increase of methanol selectivity, implying their close correlation and balance of the surface intermediates leading to the two products.

The selectivity toward HCOOCH_3_ has become increasingly noticeable at low temperatures and high-pressure operations, especially over Cu/ZnO/Al_2_O_3_. The Lewis acid sites on Al_2_O_3_ can strongly adsorb formate (HCOO*) intermediates that react further with CH_3_OH to HCOOCH_3_ (eq 1),^30^ or transiently produced formic acid through protonation of the formate may undergo esterification reaction with adsorbed CH_3_OH to produce HCOOCH_3_. The sudden drop in HCOOCH_3_ selectivity contrarily to the increased CH_3_OH and CO selectivity suggested that the HCOOCH_3_ decomposition path over Al_2_O_3_ into CH_3_OH and CO (eq 2) is likely active.^14^
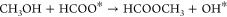
1
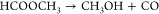
2

Considerable amounts of H_2_O formed during the reaction can activate hydrophilic Al_2_O_3_ and create Brønsted acid sites (e.g., surface hydroxyl groups) that promote HCOOCH_3_ decomposition. On the other hand, weaker Lewis acidic sites and less hydrophilicity of TiO_2_ compared to Al_2_O_3_^25^ result in generally lower HCOOCH_3_ selectivity over Re/TiO_2_. A closer look into the CO formation profile of Re/TiO_2_ shows that it behaves similarly or rather identically to HCOOCH_3_, indicating a different mechanism for HCOOCH_3_ formation/decomposition or the CO formation pathway for the two catalysts. The initial high selectivity to HCOOCH_3_ and its unstable formation profile are interesting by themselves. Based on the observations and also the previous studies aiming at HCOOCH_3_ synthesis where methanol adsorption is found rate-limiting and the support, especially its perimeter with an active metal, plays decisive roles, the amount of surface methanol and thus the coverage of methanol on the catalyst surface are important for the formation of HCOOCH_3_.^30,31^ During the initial phase of the reaction, the concentration of adsorbed methanol is expected to vary drastically where formed methanol reacts immediately with reactive formates/formic acid on the catalyst surface, but at a certain point, this will reach a steady value and this may decrease the amount of reactive formates/formic acid, explaining the formation profile of HCOOCH_3_.

The catalyst deactivation mechanism along with changes in product selectivity require further investigation in future studies. Although low reaction temperature can alleviate deactivation due to sintering, serious deactivation likely due to H_2_O under high pressure could be the next obstacle to catalytic stability.

Nevertheless, the catalytic performance of Re/TiO_2_ was more stable at 56 bar as shown in Figure S2. Overall and importantly, Re/TiO_2_ exhibits superior activity for CH_3_OH production to Cu/ZnO/Al_2_O_3_ at a low temperature, and different activation mechanisms are indicated from the product selectivity and their temporal profiles.

### 2.2 Effect of Re Loading

**Figure 2:**
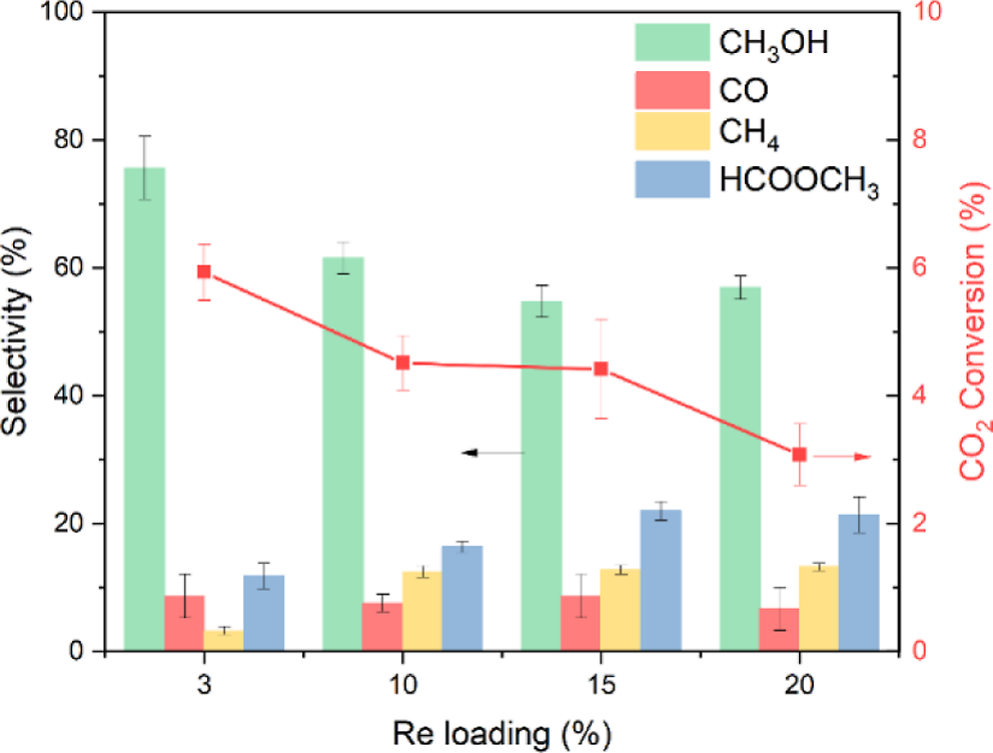
Effect of the Re loading (wt %) of Re/TiO_2_ in CO_2_ hydrogenation. H_2_/CO_2_ = 3, *T* = 150 °C, *P* = 331 bar, GHSV = 2000 h^–1^ (2 NL g_cat_^–1^ h^–1^), and TOS = 15 h.

In the previous study on Re/TiO_2_, a sub-nanometer size of Re was found important for high CH_3_OH selectivity, whereas larger Re clusters favored CH_4_ formation by the Re loading study in a batch reactor.^19^ Similar structure sensitivity was also observed over Re/In_2_O_3_.^17,18^Figure 2 confirms the finding and shows that a higher Re loading does not have a positive impact on the catalytic performances, decreasing both CO_2_ conversion and CH_3_OH selectivity. These trends are uncommon and show the strikingly high structure sensitivity or the presence of a highly reactive active site to form methanol when Re is highly dispersed.

Unlike the batch reactor with 1,4-dioxane solvent in previous work, HCOOCH_3_ was also observed and influenced by the Re loading in a continuous fixed-bed reactor. Larger Re cluster size due to increased Re loading promotes not only CH_4_ but also HCOOCH_3_ selectivity instead of CH_3_OH selectivity. CH_4_ selectivity reaches a plateau above 10 wt %, and further increase of the Re loading only resulted in the promotion of HCOOCH_3_ selectivity. Interestingly, CO selectivity appears to be independent of the Re loading. This independence of the HCOOCH_3_ and CO formation amount may indicate that the latter, as reported for Cu/Al_2_O_3_,^14^ is not formed by the decomposition of the former on Re/TiO_2_ or that the formation of CO takes place at a specific site, which is not Re-loading-dependent.

A previous study indicated that highly dispersed Re particles having oxidation states higher than Re^0^ and below Re^4+^ (ReO_2_) are beneficial in achieving high CH_3_OH selectivity.^19^ The results of this study once again show that higher Re loading has negative effects and larger Re particles accelerate both CH_4_ and HCOOCH_3_ formation. Most importantly, the Re loading affects the active sites and reaction pathways, which needs to be elucidated under *operando* conditions.

### 2.3 Unique Structures of Re/TiO_2_: *In Situ* Characterization of Fresh and Reduced Catalysts

Re is well known to alter its oxidation state in a wide range and TiO_2_ is also known for its redox properties.^21,32,33^ The combination of Re and TiO_2_ yields the uniquely active catalyst and the reducible/oxidizable nature of these may be important for the creation of the active sites or inducement of specific reaction pathways. Re would be dispersed over TiO_2_ after calcination, as shown in the elemental mapping (Figure S3). To gain deeper insights, the structure of fresh and reduced Re/TiO_2_ was characterized *in situ* using XAS and Raman spectroscopy to follow the oxidation states of Re and structural changes/disorder of the TiO_2_ lattices, respectively.

**Figure 3:**
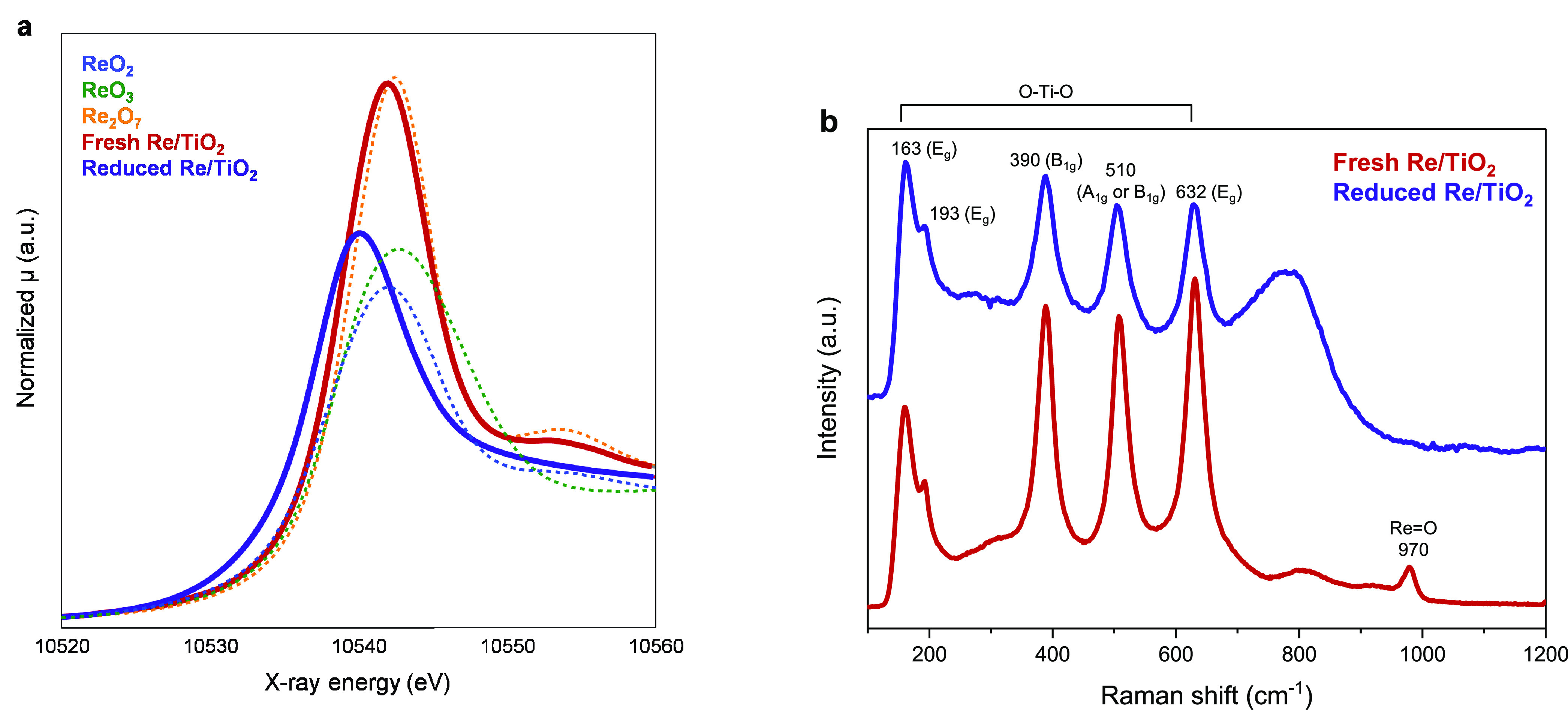
*In situ* characterization of the 3 wt % Re/TiO_2_ catalyst before and after reduction with H_2_ at 500 °C, atmospheric pressure: (a) *in situ* Re L_3_-edge XANES spectra and (b) *in situ* Raman spectra.

The Re L_3_-edge X-ray absorption near-edge structure (XANES) spectra of Re/TiO_2_ before and after reduction are shown in Figure 3a. The white line intensity and position of the calcined (fresh) catalyst at 10,542.0 eV have decreased and shifted toward lower energy of 10,540.1 eV after reduction with H_2_ at 500 °C for 0.5 h, indicating the reduction of Re_2_O_7_ species. Determining the precise oxidation states of reduced Re is challenging since the white line intensity is also influenced by the cluster size of Re.^22^ The relatively low loading of Re (3 wt %) and high XAS measurement temperature would render an extended X-ray absorption fine structure (EXAFS) analysis difficult. In addition, as can be seen in the high-angle annular dark-field scanning transmission electron microscopy (HAADF-STEM) images, the Re species are highly dispersed and heterogeneous. These features should render an EXAFS analysis particularly difficult. Note that we found that Re/TiO_2_ with 5 wt % Re loading contains features associated with both Re–O and Re–Re bonds.^34^ The coordination numbers of the Re–O and Re–Re bonds were determined to be 2.7 and 3.4, respectively.

On the other hand, the Raman spectra of the fresh Re/TiO_2_ catalyst (Figure 3b) have shown a peak at ca. 970 cm^–1^, which is attributed to ν_s_ (Re=O) of highly oxidized Re species (isolated hydrated Re_2_O_7_ species).^35^ There are no other peaks of Re=O bonds from the reduced catalysts, suggesting that most Re_2_O_7_ particles were transformed into more reduced ReO_*x*_. The peak positions at 163 (E_g_), 193 (E_g_), 390 (B_1g_), 510 (A_1g_ or B_1g_), and 632 (E_g_) cm^–1^ are assigned to the vibrational modes of the lattice and O–Ti–O bonds of the anatase structure. The peak position of TiO_2_ remains the same after reduction, suggesting no change in the anatase phase after reduction at 500 °C. The emergence of a new broad peak at 810 cm^–1^ after H_2_ exposure can either indicate the surface structural changes due to the formation of oxygen vacancies^36,37^ or the formation of ν_s_(Re–O) of Ti–O–Re or Re–O–Re bridging oxygen within the Re_*x*_O_*y*_ clusters.^38^ Although the origin of the broad peak cannot be understood by this study, the increase in Ti–O–Re coordination was expected because Re/TiO_2_ showed the tendency to form single atoms Re and sub-nanoclusters after reduction.^39^ The same observation was made in this study, in which Re disperses into more single atoms after reaction (vide infra), and the broad peak may indicate the atomic dispersion of Re into the TiO_2_ surface.

### 2.4 Structural Changes and Temporal Evolution of Surface Species during CO_2_ Hydrogenation over Re/TiO_2_

CO_2_ hydrogenation over Re/TiO_2_ under working conditions of 150 °C and 10 bar was investigated using *operando* XAS, Raman, and DRIFTS. The Re L_3_-edge XANES before and during steady-state reaction showed a slight increase in the white line intensity with a minor shift by ca. 0.2 eV (Figure S4a). These changes indicate reoxidations of Re by CO_2_ and/or redispersion of the Re cluster. More detailed results and discussion are given later. Raman spectroscopy (Figure S4b) showed no shift in peak positions but a decreased baseline, which is only related to the reduced and oxidized state of the TiO_2_ surface.

**Figure 4:**
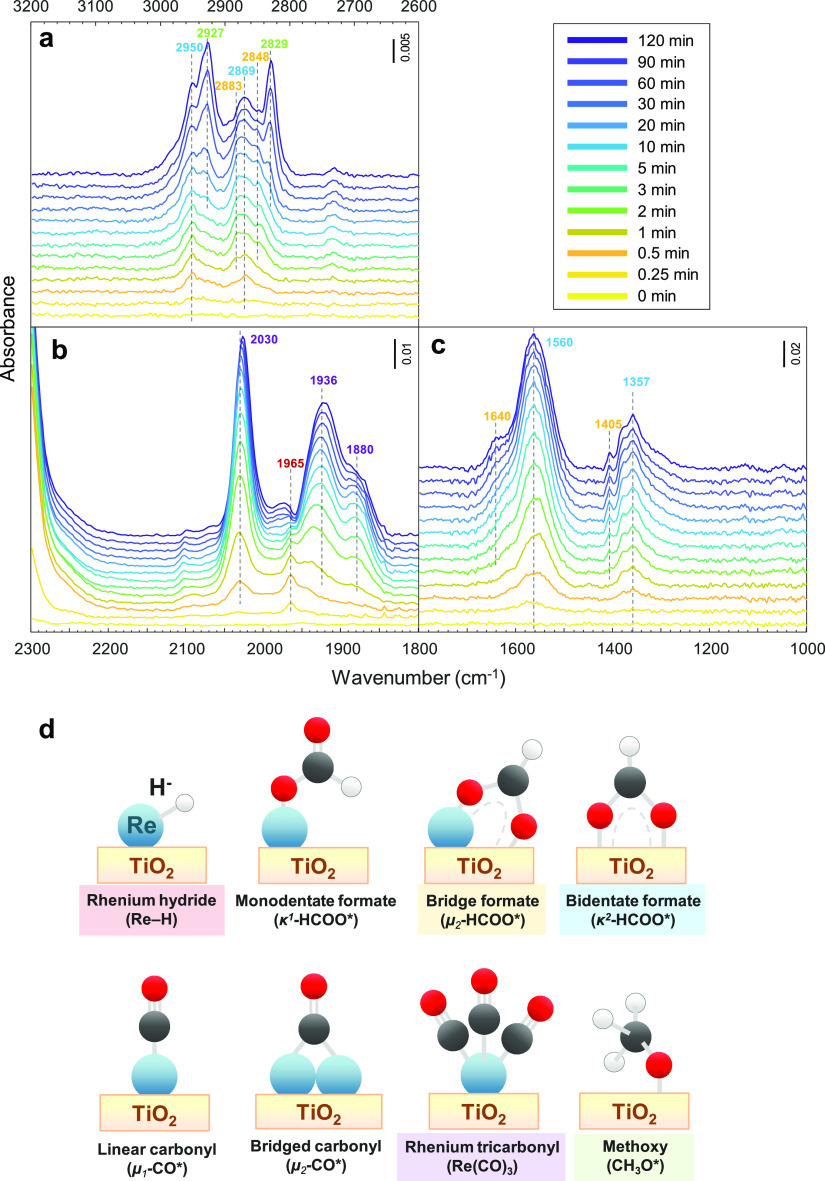
Temporal evolution of surface species obtained by *operando* DRIFTS during the initial phase of the reaction with H_2_ + CO_2_ over pre-reduced 3 wt % Re/TiO_2_. (a) ν(C–H) region, (b,c) ν(C–O) region, and (d) structures of relevant surface species. Reaction conditions: 10 mg catalyst, H_2_/CO_2_ = 3, *T* = 150 °C, *P* = 10 bar, *F*_total_ = 10 NmL min^–1^.

Time-resolved DRIFTS provides insights into the temporal evolution of surface species during CO_2_ hydrogenation over the freshly reduced catalyst (Figure 4). Identified surface species are kinetically distinguishable, which facilitates the identification and assignment of each IR band. Table S1 summarizes the peak assignments from the literature for each surface species and IR vibrational modes. CO_2_ hydrogenation using D_2_ (^2^H_2_) helps us to identify surface species containing H atoms, e.g., through C–H and O–H bonds due to the shift in the vibrational frequencies (Figure S5). Moreover, kinetic isotope effects (KIEs) can alter the intermediate/product formation rates, and one can learn about rate-limiting steps.^11,12^ On the other hand, ^13^CO_2_ hydrogenation helps us to identify the species containing the C=O bond (Figure S6). For example, in this study, both D_2_ and ^13^CO_2_ played a crucial role in the identification of rhenium hydride (Re–H), which are obscured by carbonyls (CO*).

The ν(C–H) bands in the 2500–3200 cm^–1^ region are presented in Figure 4a, while ν(C–O) bands in 1000–1800 and 1800–2300 cm^–1^ regions are shown in Figure 4b,c. The structures of relevant surface species are illustrated in Figure 4d. The characteristic IR bands of rhenium hydride (Re–H, 1965 cm^–1^) and bridging or chelating bidentate formate on TiO_2_ (κ^2^-HCOO*, 2950, 2869, 1560, and 1357 cm^–1^) appear immediately after the reaction was initiated. Linear carbonyl species (μ_1_-CO*, 2030 cm^–1^) appear after 0.5 min. Bridge carbonyls (μ_2_-CO*, 1936 and 1880 cm^–1^) appear after 2 min. However, we later confirm that the bands identified as μ_1_-CO* and μ_2_-CO* are the parts of the rhenium tricarbonyls complex [Re(CO)_3_] (vide infra). The bands that appeared simultaneously at 2883, 2848, 2732, 1640, and 1405 cm^–1^ can be assigned to adsorbed methyl formate (HCOOCH_3_*)^40,41^ or adsorbed formic acid (HCOOH*).^42−44^ However, those adsorbed molecules were not usually observed under *operando* DRIFTS since they are prone to be chemically adsorbed in the form of κ^2^-HCOO*.^30,31,45^ These bands were later identified as monodentate formate on Re (κ^1^-HCOO*) or bridging formate (μ_2_-HCOO*) over Re or the Re–O–Ti interface.^46^ Methoxy species (CH_3_O*, 2829, and 2927 cm^–1^) appear in the latest order after 10 min of reaction.

In summary, the surface species temporally evolved in the following order: κ^2^-HCOO* = Re–H (0.25 min) → μ_1_-CO* (0.5 min) → μ_2_-CO* (Re(CO)_3_ formation) = μ_2_-HCOO* (2 min) → CH_3_O* (10 min). It should be highlighted that a faster spectral acquisition than 10 s is required to distinguish the formation rate between κ^2^-HCOO* and Re–H.

An identical experiment was performed over bare TiO_2_ to understand the role of TiO_2_ support (Figure S8). In the absence of Re metal, only monodentate carbonate (κ^1^-CO_3_*, 1369, and 1583 cm^–1^), monodentate bicarbonate (κ^1^-HCO_3_*, 1429, and 1656 cm^–1^), bidentate bicarbonate (κ^2^-HCO_3_*, 1223, 1503, and 1618 cm^–1^), and weakly adsorbed carbon dioxide (CO_2_*, 1334 cm^–1^) form over TiO_2_. There is no signal of C–H bonds in the 2500–3200 cm^–1^ region, confirming that formate cannot be formed without Re. CO_3_* and HCO_3_* could form with CO_2_ via lattice oxygen and the surface hydroxy group (Ti–OH), respectively. Additionally, no traces of CO* were detected since coordination to Re atoms is required for CO* species.

The formation of carbonyl species over Re/TiO_2_ during the reaction (Figure 4b) indicates the reduction of the Re=O bond, which is generally required to form a coordination complex with CO. The tentatively assigned μ_1_-CO* and μ_2_-CO* form with distinct kinetics. However, the formation of bridge carbonyls is less favored or not possible on atomically dispersed Re due to the lack of adjacent Re atoms. At the reaction temperature, the adsorption of CO can lead to disruption and dispersion of Re crystallites, which indeed takes place during the reaction as described later and eventually leads to the formation of rhenium carbonyl complexes like Re(CO)_3_.^47^ Here, based on the spectral features, the carbonyl species are assigned to Re(CO)_3_, although the number of carbonyls can fluctuate depending on the coordinating groups and environment around Re.

The involvement of Re–H (1965 cm^–1^) in the HCOO* formation is confirmed by both ^13^CO_2_ hydrogenation (Figure S6) and CO_2_ hydrogenation using D_2_ (^2^H_2_) (Figure S5). Since the Re–H band is usually overlapping with that of CO*, the red shift of all CO* species bands using ^13^CO_2_ can reveal an unaffected Re–H vibrational band. On the other hand, the consumption of Re–H (formed during reduction) was revealed by the D_2_ exchange, causing the red shift of Re–D to 1355 cm^–1^ (overlapping with κ^2^-HCOO*). However, the role of Re–H in HCOO* formation remains ambiguous: through hydrogenation of CO_3_* and/or HCO_3_* on TiO_2_^48^ or direct activation of CO_2_ via hydride transfer over the Re atom.

**Figure 5:**
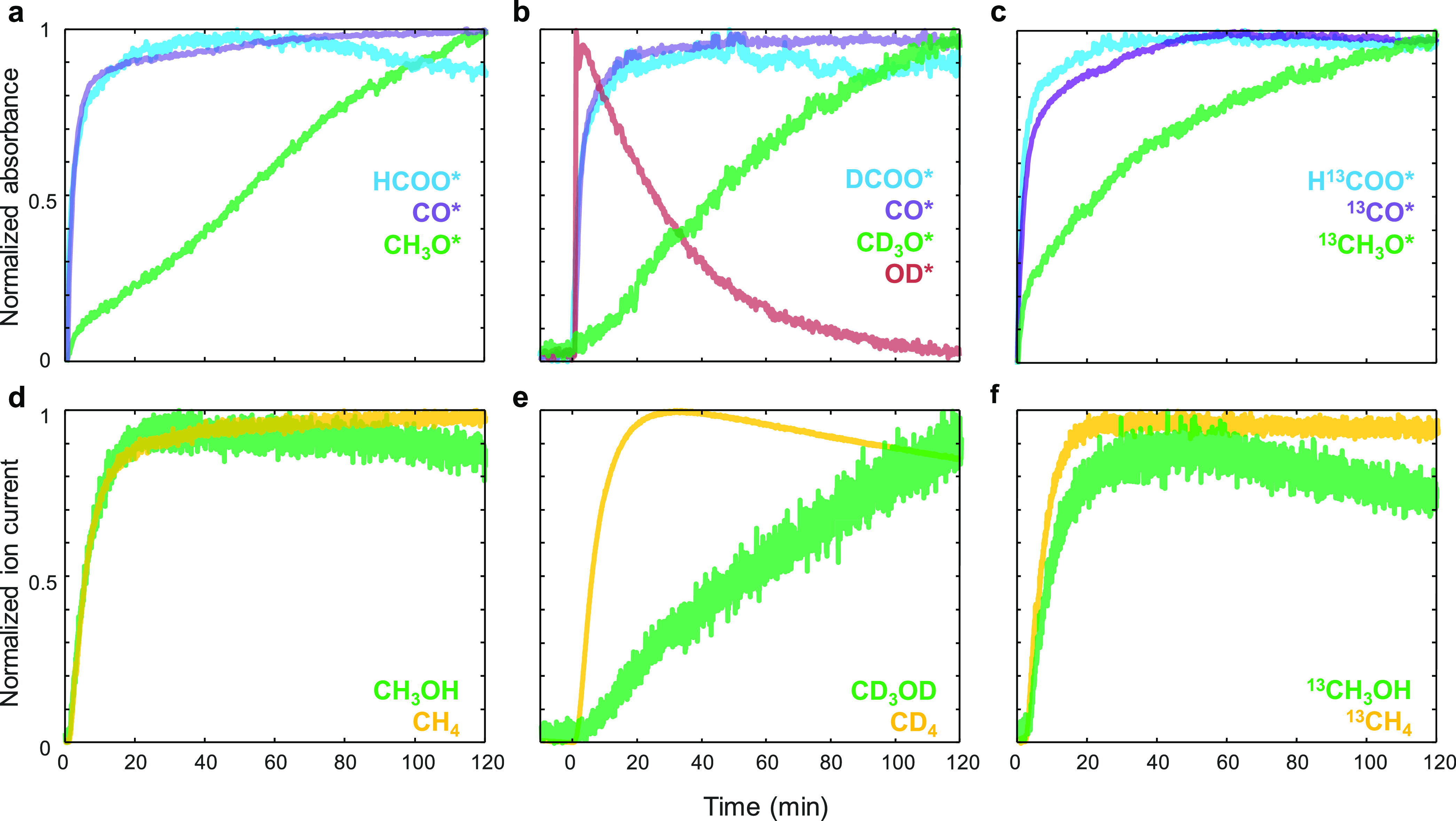
Temporal evolution of normalized absorbance of the main surface species obtained from *operando* DRIFTS during reaction with (a) H_2_ + CO_2_, (b) D_2_ + CO_2_, and (c) H_2_ + ^13^CO_2_ over pre-reduced 3 wt % Re/TiO_2_. Corresponding normalized ion current signals of methanol and methane obtained with a mass spectrometer during reaction with (d) H_2_ + CO_2_, (e) D_2_+CO_2_, and (f) H_2_ + ^13^CO_2_ over 3 wt % Re/TiO_2_. Wavenumber (cm^–1^): κ^2^-HCOO* (1565), μ_1_-CO* (2026), CH_3_O* (2830), κ^2^-DCOO* (2181), μ_1_-CO* (2026), CD_3_O* (2063), OD* (2704), κ^2^-H^13^COO* (1527), μ_1_-^13^CO* (1976), ^13^CH_3_O* (2827). The MS of CD_4_ at *m*/*z* = 18 is shown instead of *m*/*z* = 20 because of overlapping with D_2_O and H_2_O contribution was assumed to be negligible.

The normalized IR bands of surface species and mass spectrometry (MS) signals of gaseous products are compared in Figure 5. During the CO_2_ hydrogenation, CH_3_OH formation (Figure 5d) reaches a steady state within 20 min, and its profile is similar to that of κ^2^-HCOO* formation (Figure 5a). In contrast, CH_3_O* formation has shown a significant delay in its increase on the catalyst surface. This suggests that the observed CH_3_O* is not required as the intermediate for CH_3_OH formation. ^13^CO_2_ hydrogenation yielded a temporal evolution of surface and gaseous species similar to the case of ^12^CO_2_ (Figure 5c,f).

Furthermore, an identical isotopic labeling experiment was performed using D_2_ instead of H_2_. During the CO_2_ hydrogenation with D_2_, a similar κ^2^-DCOO* (2180 cm^–1^) formation profile to that of HCOO* is observed, suggesting that CO_2_ activation to κ^2^-HCOO* is not affected by the KIE (Figure 5b). However, the CD_3_OD formation (Figure 5e) is much slower compared to CH_3_OH formation (Figure 5d), while CD_4_ is virtually unaffected (Figure 5e). This indicates completely different formation mechanisms and involved intermediates to form methanol and methane. The CD_3_OD signal shows a similar profile to that of CD_3_O* (2061 cm^–1^), which behaves in an opposite manner to that of the terminal deuteroxyl group (OD*, 2704 cm^–1^). To understand the location and roles of this CD_3_O* as well as the correlation between CD_3_O* and OD*, a CH_3_OH adsorption experiment was performed, followed by titration using D_2_ (Figure S8). After CH_3_OH was adsorbed as CH_3_O*, titration of CH_3_O* by D_2_ produced CH_3_OD and regenerated OD* similar to the inverted relationship between CD_3_O* and OD*. This suggested that the observed CH_3_O* species are located on TiO_2_ support and originated from CH_3_OH adsorption over the Ti–OH sites. A similar experiment is performed over TiO_2_ support (Figure S9). However, the Ti-OD at 2704 cm^–1^ was not observed, which indicates that the formation of the Ti–OH group via heterolytic dissociation of H_2_ is not possible over TiO_2_, at least at this temperature. This confirms the role of Re for H_2_ dissociation and hydride transfer over TiO_2_ via H-spillover.^49^ Moreover, the CD_3_O* is unreactive to D_2_ without Re. From the above results, it is clear that CD_3_OD produced during CO_2_ hydrogenation (with D_2_) reacted with OD* to form CD_3_O*. Therefore, the slower product formation rate due to CD_3_OD adsorption led to delayed detection of CD_3_OD compared to CD_4_.

The instantaneous formation of OD* during CO_2_ hydrogenation with D_2_ suggests that D-spillover from Re is rapid within the time-scale of the experiment compared to the consumption of Re–H (produced during catalyst activation via reduction with H_2_) to form DCOO*. The gradual decline of the OD* concentration discarded the Ti–OH role in κ^2^-HCOO* formation via CO_2_ activation into HCO_3_* and its subsequent hydrogenation at low temperatures since κ^2^-HCOO* saturation on the surface was significantly faster than OD* consumption. Notably, the quickly formed OD* on TiO_2_ is gradually replaced by CD_3_O*. This gradual surface species evolution might be linked to the initial selectivity changes and consequent HCOOCH_3_ formation during the catalytic tests (Figure 1).

In addition, CO hydrogenation was also carried out to understand the mechanistic differences to CO_2_ hydrogenation, as shown in Figure S10. Compared with CO_2_ hydrogenation, similar Re(CO)_3_ (2029, 1915, and 1873 cm^–1^) are observed. The gaseous CO (2169 cm^–1^) and additional peaks of Re_2_(CO)_10_ (2104 and 1995 cm^–1^) also appear. Only trace amounts of adsorbed H_2_O, κ^2^-HCOO*, and CH_3_O* are observed, which indicated the lack of CH_3_OH formation.^19^ There is no indication of formyl (HCO*) and formaldehyde (H_2_CO*) produced via stepwise hydrogenation of carbonyls as the main intermediate for CO hydrogenation.^50^ The formate species, which is a more natural intermediate in CO_2_ hydrogenation via hydride transfer to CO_2_, seems indeed the key intermediate in producing methanol over Re/TiO_2_. The roles of HCOO*, CH_3_O*, and CO* were further investigated using D_2_ and ^13^CO_2_ SSITKA.

### 2.5 Unveiling the Distinct Pathways toward CH_3_OH and CH_4_ Using SSITKA

SSITKA-DRIFTS is a powerful technique that combines transient isotopic exchanges and *operando* surface species responses while maintaining the characteristics of the steady-state operation keeping the constant partial pressure of reactants. The time-resolved DRIFT spectra containing the kinetically separable and isotopically labeled surface species were analyzed using multivariate spectral analysis, more precisely multivariate curve resolution (MCR),^51,52^ to obtain their kinetically pure spectra and corresponding concentration profiles. Furthermore, the surface species concentration will be correlated with the concentration of gaseous products detected by MS to extract mechanistic information. To avoid misinterpretation, it should be noted that we show the MS signals of the isotopic products wherein the *m*/*z* are not overlapping. For example, the abundant signals of CD_4_ at *m*/*z* 20 and 18 overlap with D_2_O and H_2_O, respectively.

**Figure 6:**
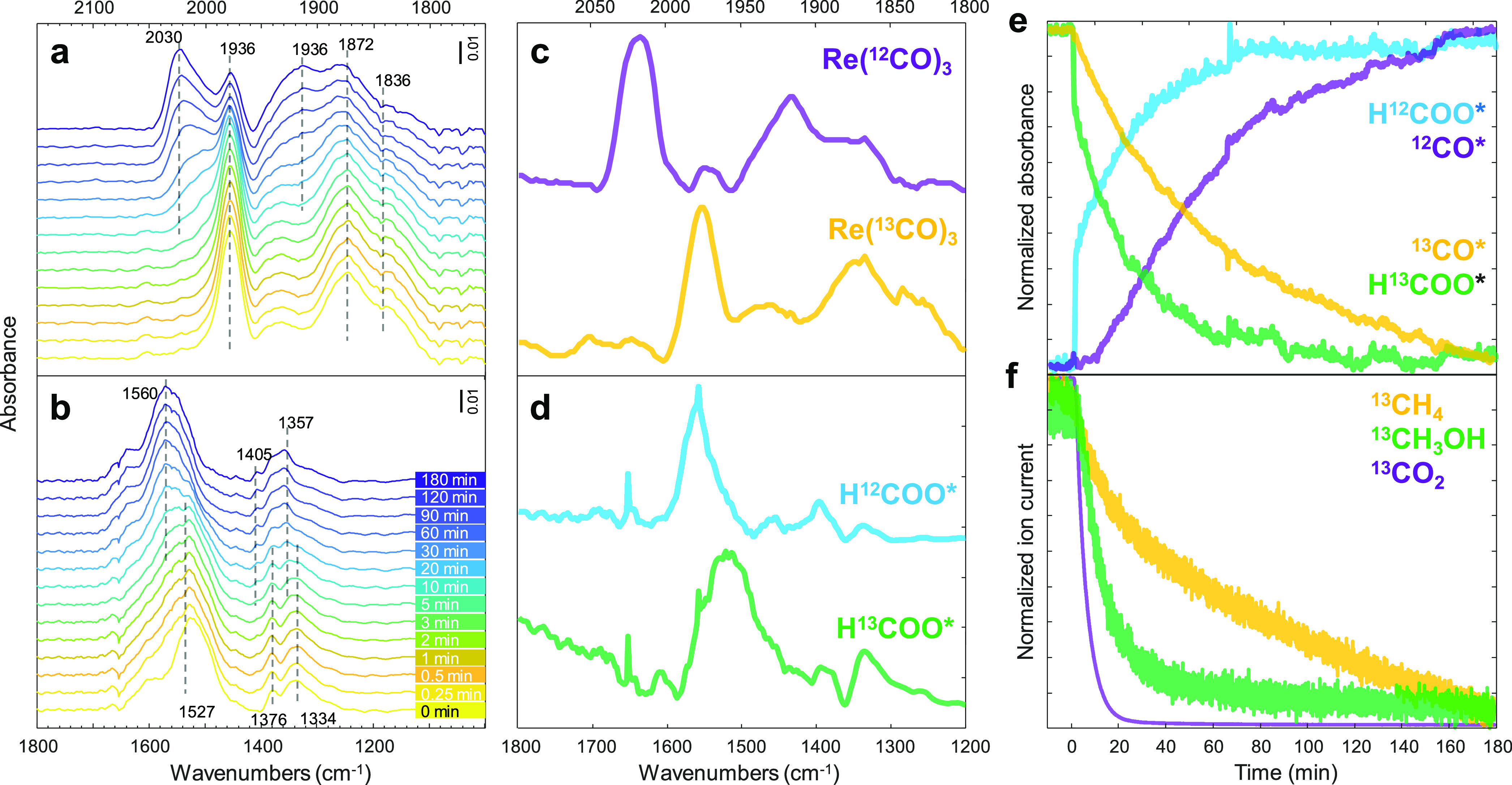
Transient responses of surface species and gas products during the steady-state isotopic switching from ^13^CO_2_ + H_2_ to ^12^CO_2_ + H_2_. Time-resolved DRIFT spectra of (a) ^12^CO* and ^13^CO* and (b) H^12^COO* and H^13^COO*. (c,d) Components’ spectra obtained by MCR applied on the time-resolved DRIFT spectra. (e) Concentration profiles of the spectra of the corresponding components obtained by MCR. (f) Corresponding normalized ion current signals of isotope-labeled products. *The formation of ^12^CH_4_ and ^12^CH_3_OH is not shown due to the contribution from ^13^CH_4_ and ^13^CH_3_OH. Reaction conditions: 10 mg catalyst, H_2_/CO_2_ = 3, *T* = 150 °C, *P* = 10 bar, *F*_total_ = 10 NmL min^–1^.

The roles of formate and carbonyls can be investigated by switching from the ^13^CO_2_ + H_2_ to the ^12^CO_2_ + H_2_ stream. As shown in Figure 6a,b, ^13^CO* (1936, 1872, and 1836 cm^–1^) and κ^2^-H^13^COO* (1527 and 1334 cm^–1^), formed during the steady-state ^13^CO_2_ hydrogenation, disappeared after isotopic switching, leading to the formation of ^12^CO* (2030, 1936, and 1880 cm^–1^) and κ^2^-H^12^COO* (1560 and 1357 cm^–1^), respectively. The overlapping bands could be deconvoluted by MCR. Especially, if the concentrations of surface species are kinetically distinguishable, MCR enables us to obtain a chemically pure spectrum of each surface species. The MCR-resolved spectra of κ^2^-HCOO* (Figure 6d) show two distinguishable characteristics of κ^2^-H^12^COO* and κ^2^-H^13^COO*. On the other hand, the MCR-resolved spectra of CO* show three bands with the same kinetic response, confirming the formation of Re(CO)_3_ complexes (Figure 6c) with kinetically indistinguishable carbonyls on this time scale.^47^

The MCR-resolved concentration profiles of CO* and HCOO* are shown in Figure 6e. The symmetrical responses between ^13^C- and ^12^C-containing species after isotopic switching are observed. The consumption/formation of κ^2^-HCOO* is noticeably faster than that of Re(CO)_3_, indicating higher reactivity of κ^2^-HCOO* toward hydrogenation. Moreover, the Re(^12^CO)_3_ formation had shown a significant delay compared to κ^2^-H^12^COO* after isotopic switching. This indicates that κ^2^-HCOO* could be the intermediate for CO* via κ^2^-HCOO* decomposition^53^ and eventually transforms into Re(CO)_3_ complexes. This finding is consistent with the result from the temporal evolution experiment, in which Re–H and HCOO* were the first species to form (vide supra).

In the gas phase, the ^13^CH_3_OH response decays faster than ^13^CH_4_ (Figure 6f), which is congruent to the faster consumption rate of κ^2^-H^13^COO* compared to ^13^CO* (Figure 6e). This suggested the different pathways of CH_3_OH and CH_4_ formation: CH_3_OH via direct hydrogenation of κ^2^-HCOO* and CH_4_ via CO* formation and subsequent hydrogenation. It should be noted that there was no noticeable change in the CH_3_O* profile detected from ν(C–H) upon switching from the ^13^CO_2_ + H_2_ to the ^12^CO_2_ + H_2_ stream, which suggested no correlation between CH_3_O* and CH_4_.

**Figure 7:**
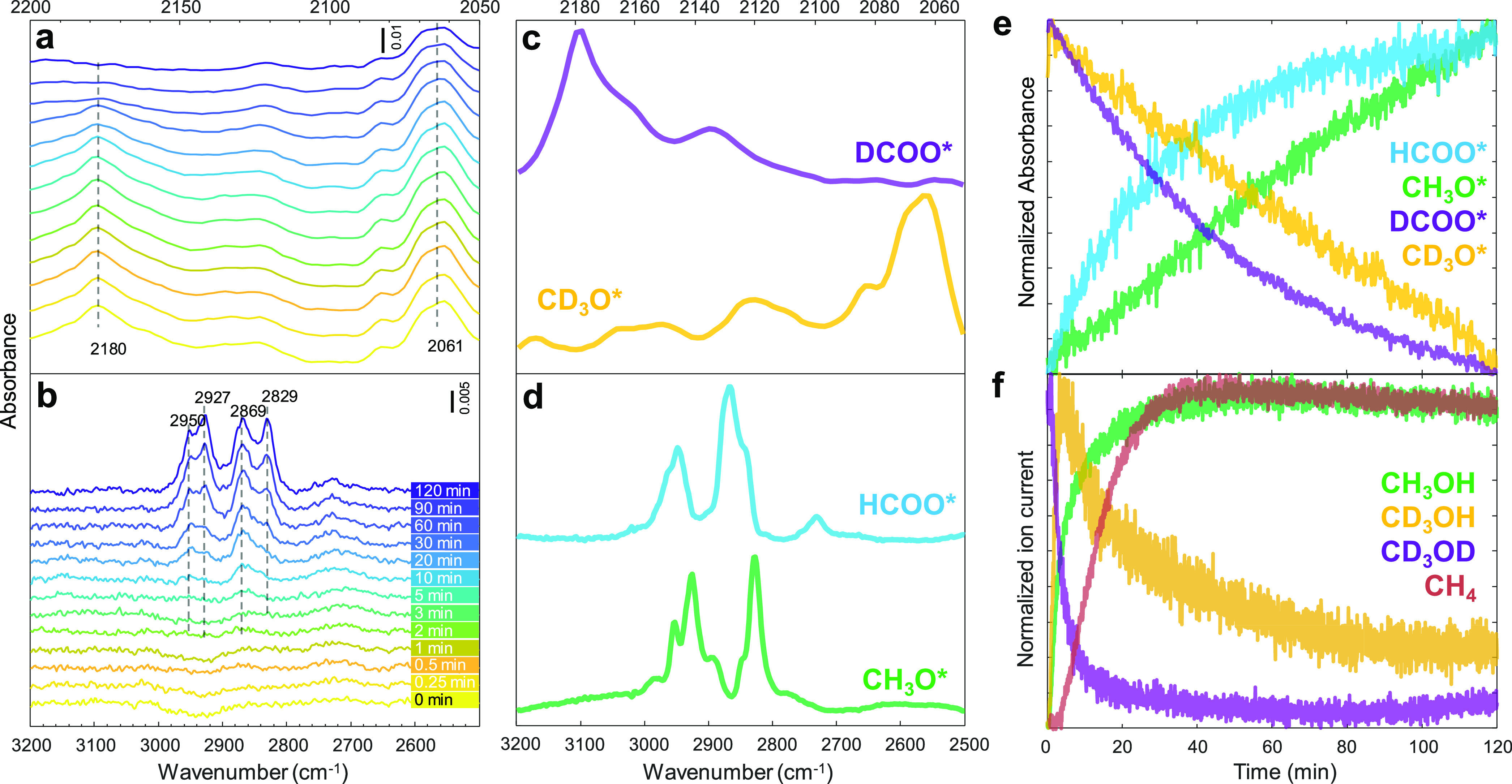
Transient responses of surface species and gas products during the steady-state isotopic switching from CO_2_ + D_2_ to CO_2_ + H_2_. Time-resolved DRIFT spectra of (a) DCOO* and CD_3_O* and (b) HCOO* and CH_3_O*. (c,d) Components’ spectra obtained by MCR applied on the time-resolved DRIFT spectra. (e) Concentration profiles of the spectra of the corresponding components obtained by MCR. (f) Corresponding normalized ion current signals of isotope-labeled products. Reaction conditions: 10 mg catalyst, H_2_(or D_2_)/CO_2_ = 3, *T* = 150 °C, *P* = 10 bar, *F*_total_ = 10 NmL min^–1^.

Transient isotopic switching from the steady-state CO_2_ + D_2_ to the CO_2_ + H_2_ feed can shed light on the roles of κ^2^-HCOO* and CH_3_O*. As shown in Figure 7a, κ^2^-DCOO* and CD_3_O* formed during the previous steady state started to disappear after isotopic switching, leading to the formation of κ^2^-HCOO* and CH_3_O*, as shown in Figure 7b. However, the consumption/formation of κ^2^-DCOO*/κ^2^-HCOO* was noticeably faster than that of CD_3_O*/CH_3_O*. Similar profiles were observed in transient isotopic switching from steady-state CO_2_ + H_2_ to CO_2_ + D_2_ (Figures S11 and S12). The MCR provides two kinetically distinguishable spectra of κ^2^-HCOO* and CH_3_O* in the C–H stretching region and the other two spectra of κ^2^-DCOO* and CD_3_O* in the C–D stretching region (Figure 7c,d), as well as their concentration profiles (Figure 7e). The concentration profiles obtained by MCR show a symmetrical relationship between deuterated species and hydrogenated species, indicating the isotopic exchanges. CD_3_OH spiked rapidly after isotopic switching followed by a long decay (Figure 7f), indicating that the reaction between CD_3_O* and spilled-over H takes place and takes time due to the high stability of the former on TiO_2_.

Importantly, CH_3_OH is produced right after switching and reaches the steady state after 20 min regardless of the remaining κ^2^-DCOO* that disappears after 60 min and of CD_3_O* that reacts slowly and remains on the surface even after 120 min. This piece of evidence confirms that observable CH_3_O* on TiO_2_ is a spectator and not the intermediate for CH_3_OH formation. Similarly, the slowly reacted κ^2^-HCOO* on TiO_2_ could be proven as a spectator as well. However, there is the possibility that only κ^2^-HCOO* located adjacently to the Re is the active species, while those not in proximity are unreactive. Transient techniques are required to capture the short-lived surface species responsible for CH_3_OH.

### 2.6 Capturing the Surface Active Intermediates toward CH_3_OH and CH_4_ Using Transient Experiments (Concentration Modulation)

The transient experiment can utilize not only isotopes but also the drastic changes in the reactant concentration, e.g., passing only one of the reactants momentarily and allowing the detection of surface species responding to periodic perturbation. This technique can improve sensitivity and unveils spectral features that are not accessible by steady-state experiments. The transient experiment requires multiple modulation cycles for the system to reach a quasi-steady state. Such states include the oxidation state of active metal, local structure, surface species coverage, etc. The responses of those states after the quasi-steady state are similar in the following cycles, allowing cycle averaging to improve the signal-to-noise ratio.^26,27^

**Figure 8:**
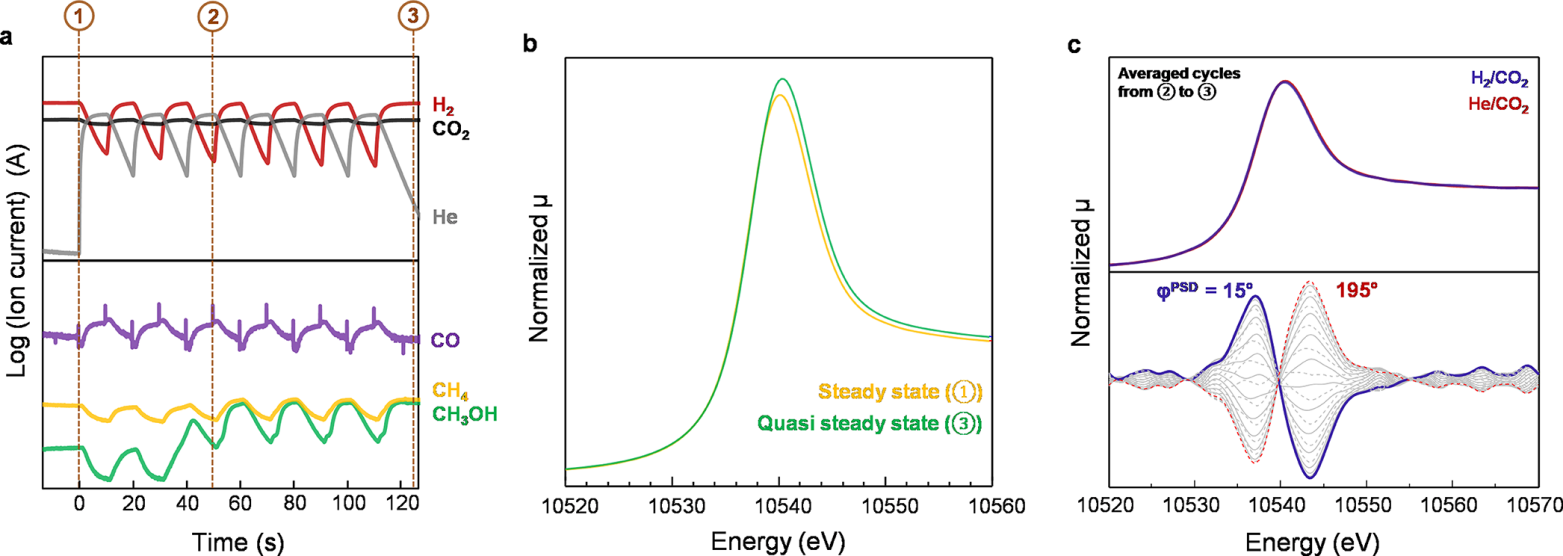
Transient H_2_ + CO_2_ and He + CO_2_ experiment. (a) Corresponding normalized ion current signals from a mass spectrometer. (b) *Operando* Re L_3_-edge XANES spectra. (c) Phase-resolved amplitude spectra from Re L_3_-edge XANES: Spectra are within φ^PSD^ = 0–360° at steps of φ^PSD^ = 15°. He balances are used in the CO_2_ phase to maintain partial pressure. Reaction conditions: H_2_/CO_2_ = 3, *T* = 150 °C, *P* = 10 bar, *F*_total_ = 10 NmL min^–1^.

The example of a quasi-steady state over Re/TiO_2_ is clearly shown in the transient experiment of the modulated flow of H_2_ + CO_2_ vs CO_2_. As shown in Figure 8a, the reaction under H_2_+CO_2_ had reached a steady state (①) before the transient experiment. Modulation with CO_2_ induced structural change of Re/TiO_2_, leading to an increase in the CH_3_OH signal in the gas phase. The structural change reached a quasi-steady state after 3 cycles (②) and remained irreversible after the transient experiment (③). The Re L_3_-edge XANES showed a notable increase in the white line intensity compared to the previous steady state (Figure 8b). During the quasi-steady state (②), the change in response to the periodic concentration change was infinitesimal, but the subtle change was confirmed by the phase-resolved spectra obtained by phase-sensitive detection (Figure 8c).^26^ The in-phase and out-of-phase positions are at 10,537 and 10543.5 eV, but they are assumed to be artificially created peaks to describe the shift of absorption peaks. Rather, this study shows that the Re redox state does change, although the extent is small, around the peak around 10,540 eV, which can be in the range of Re^0^–Re^4+^.

**Figure 9:**
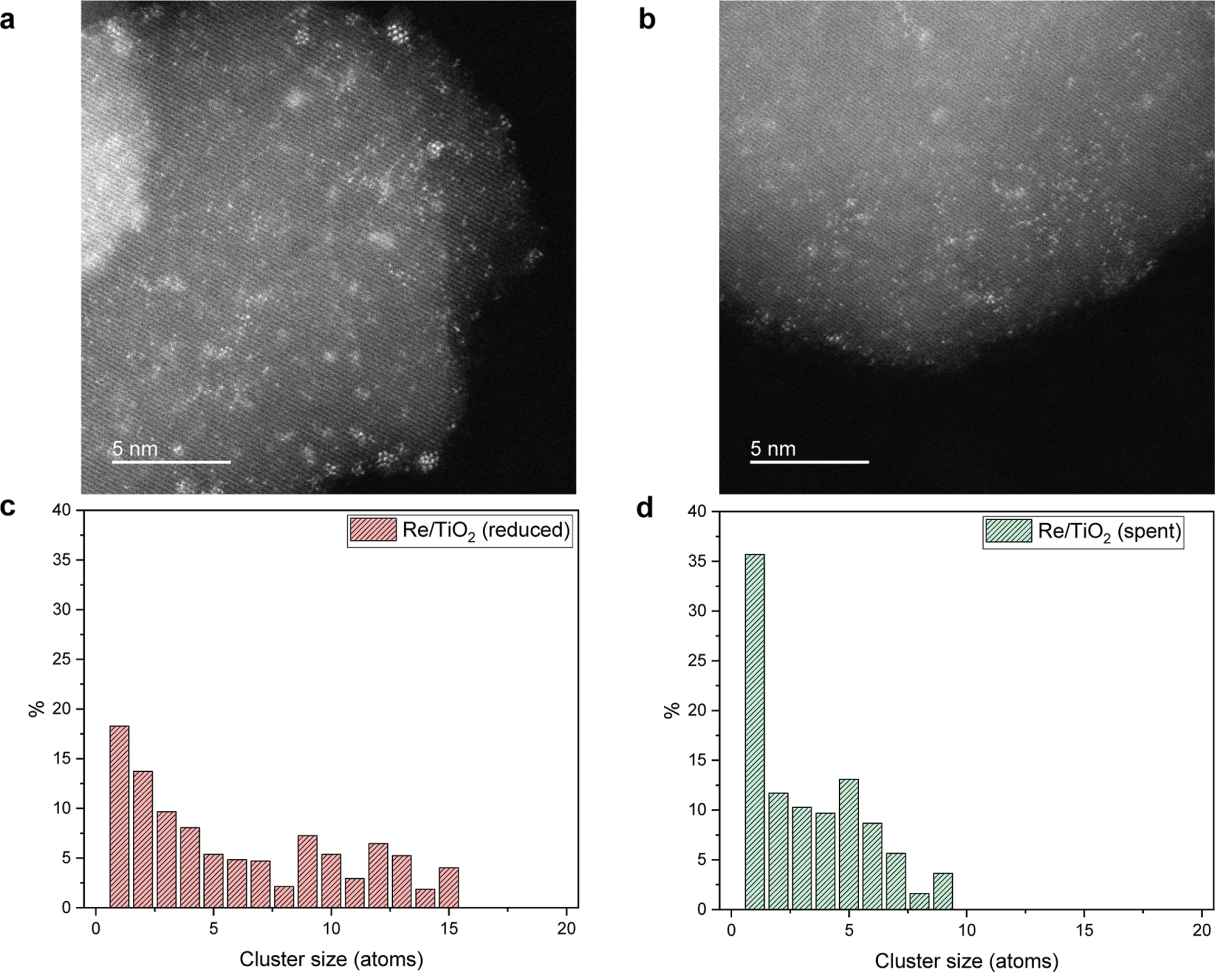
Representative HAADF-STEM images of Re/TiO_2_: (a) after reduction (500 °C in H_2_) and (b) after the transient experiment (150 °C and 10 bar). (c) Re cluster size distribution of Re/TiO_2_ was determined from HAADF-STEM: (c) after reduction (500 °C in H_2_) and (d) after the transient experiment (150 °C and 10 bar).

Representative HAADF-STEM images of reduced and spent Re/TiO_2_ (Figure 9a,b) show that increased dispersion of Re clusters is responsible for the irreversible change until reaching the quasi-steady state (①). As shown in Figure 9c,d, the particle size distribution of Re clusters becomes narrower toward single Re atoms and clusters containing a few Re atoms after the transient reaction. The decomposition and (re)dispersion of metal nanoparticles are often related to the concentration of defect sites that enthalpically stabilize adatoms.^54,55^ The Raman spectra during the transient experiment (Figure S13) show no shifts in two E_g_ modes (163 and 193 cm^–1^) toward higher frequency as an indication of oxygen vacancy over TiO_2_.^56,57^ However, the lower signal intensity during H_2_ + CO_2_ than He + CO_2_ due to the baseline shift implies the relatively oxygen-deficient TiO_2_ that may facilitate the decomposition and (re)dispersion of Re particles.

**Figure 10:**
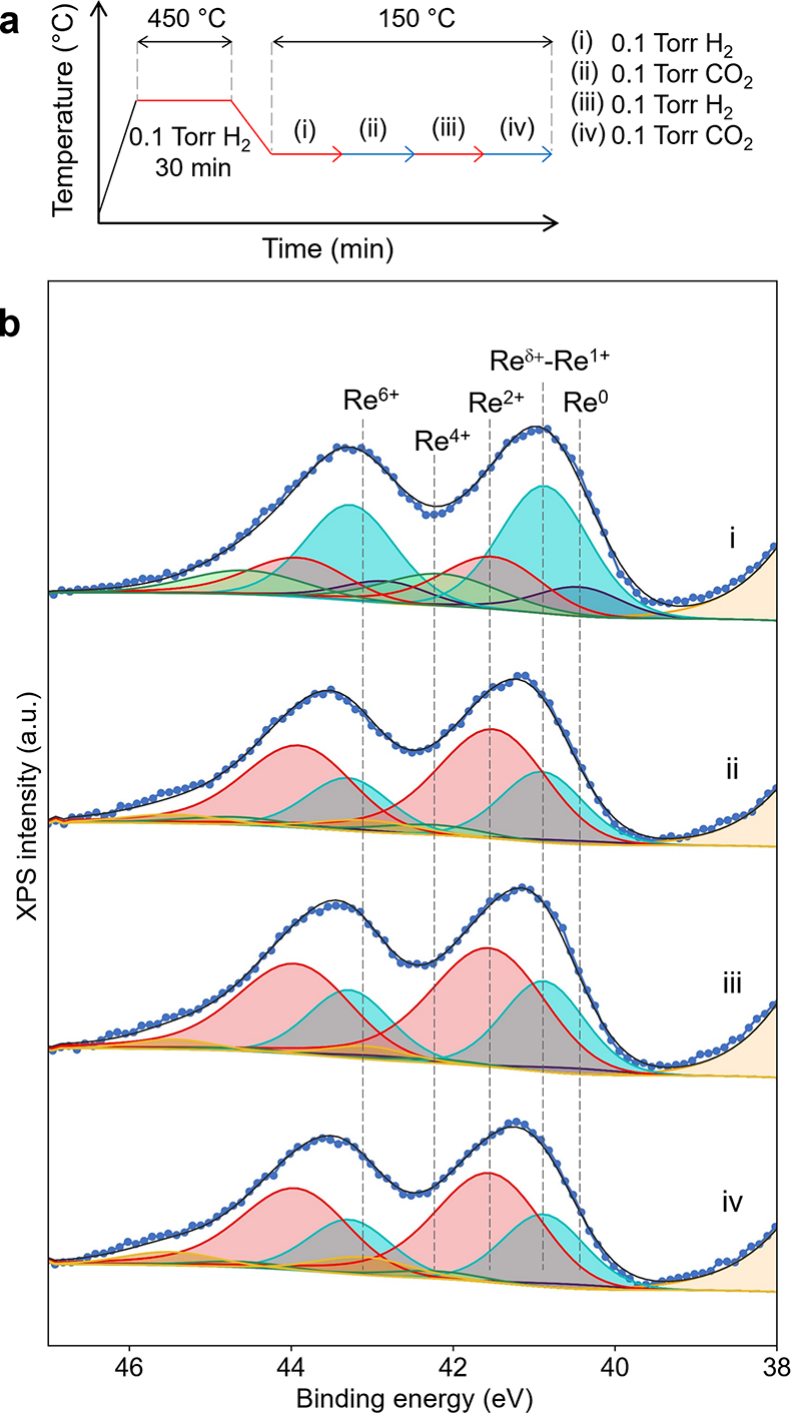
(a) Experimental conditions during the AP-XPS study. (b) Re 4f AP-XPS spectra of Re/TiO_2_ under exposure to (i) H_2_, (ii) CO_2_, (iii) H_2_, and (iv) CO_2_ at 150 °C. Blue dots: raw spectrum; black line: sum; yellow: Re^6+^; green: Re^4+^; red: Re^2+^; light blue: Re^δ+^–Re^1+^; navy: Re^0^; beige: Ti 3p.

To gain more precise insights into the redox state of Re, an AP-XPS study was performed (Figure 10). Re/TiO_2_ was reduced at 450 °C, which is lower than the standard reduction temperature for the pretreatment, due to a limitation of our experimental setup (for more detailed experimental and analysis procedures, see the Supporting Information). Re/TiO_2_ after reduction at 450 °C was found to contain multi-oxidation states of Re such as Re^0^, Re^δ+^–Re^1+^, Re^2+^, Re^4+^, and Re^6+^. Although Re^δ+^–Re^1+^ and Re^2+^ are not known to exist as a stable bulk oxide, they can exist as surface species.^32^ Note that Greiner and co-workers further divided the Re^δ+^–Re^1+^ species into subcomponents in their study.^32^ However, given our limited spectral resolution and the potential difficulties associated with interpreting an excessive number of parameters, we treated them as a unified component in our study. In addition, other species including Re^3+^, which have been reported as surface species and/or in metal complexes, can also be present although the fitting with only the aforementioned species gave a satisfactory fit in the present study. The amount of surface cationic Re species increased under a CO_2_ atmosphere at 150 °C, suggesting the oxidation of Re by CO_2_ (Table 1). Subsequent introduction of H_2_ reduced the cationic Re species, followed by reoxidation by CO_2_. These results indicate that CO_2_ oxidizes the supported Re and the oxidized Re is reduced by H_2_ reversibly, suggesting the participation of surface cationic species such as Re^δ+^–Re^1+^, Re^2+^, Re^4+^, and Re^6+^ in CO_2_ hydrogenation, while the remaining Re^0^ as well as low-valence state Re species can play role in H_2_ activation. We are aware that investigations conducted at low pressures (millibar scale) may lead to different conclusions to identify catalytic species.^5^ Nevertheless, direct observations of the surface Re species under repeated reduction (H_2_)–oxidation (CO_2_) cycles using XPS techniques can provide qualitative insights into redox behavior of the Re species.

**Table 1:** Peak Concentrations in the Re 4f AP-XPS Spectra of Re/TiO_2_ at 150 °C under Various Gas Conditions (Figure 10)

	peak concentration (%)				
gas conditions	Re^0^	Re^δ+^–Re^1+^	Re^2+^	Re^4+^	Re^6+^
(i) 0.1 Torr H_2_^a^	11.5	48.6	23.9	16.0	0
(ii) 0.1 Torr CO_2_	1.8	27.5	60.8	5.4	4.6
(iii) 0.1 Torr H_2_ (2nd)	2.5	30.9	60.6	1.7	4.3
(iv) 0.1 Torr CO_2_ (2nd)	2.2	25.8	59.5	6.5	6.1

a Measured at 150 °C right after the H_2_ reduction pretreatment at 450 °C.

**Figure 11:**
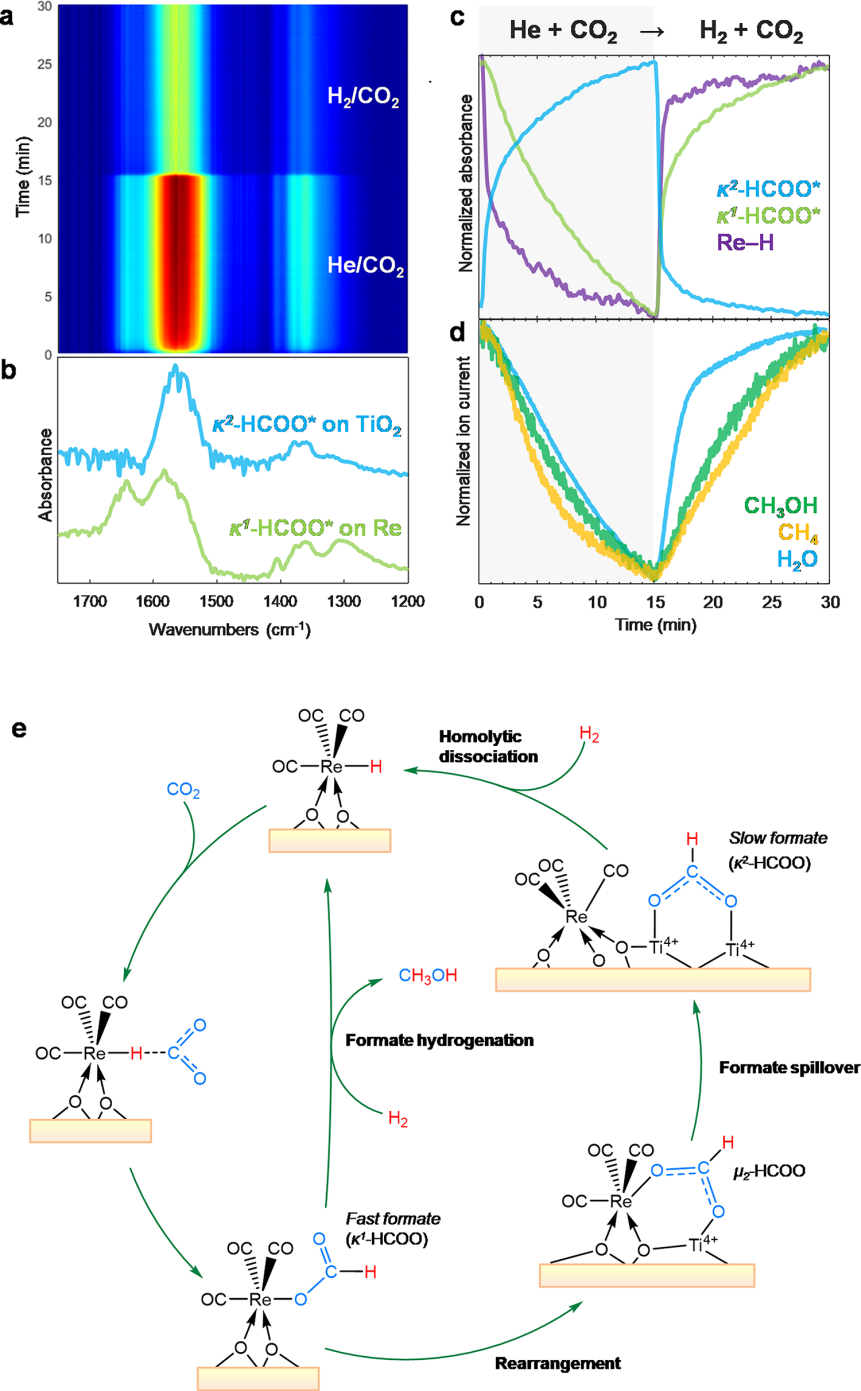
Transient DRIFTS study on CO_2_ hydrogenation over a 3 wt % Re/TiO_2_ catalyst. (a) Time-resolved DRIFT spectra upon transient concentration perturbation using H_2_ + CO_2_ and He + CO_2_ at 150 °C and 10 bar. (b) Components’ spectra obtained by multivariate spectral analysis applied on the time-resolved DRIFT spectra. (c) Concentration profiles of the corresponding components’ spectra obtained by the multivariate spectral analysis. (d) Corresponding normalized ion current signals obtained from MS. (e) Proposed mechanism for CO_2_ activation to monodentate formate. Possible dihydride species are shown as monohydride. Reaction conditions: 10 mg catalyst, H_2_/CO_2_ = 3, *T* = 150 °C, *P* = 10 bar, *F*_total_ = 10 NmL min^–1^.

The transient DRIFT spectra obtained by alternatingly passing H_2_ + CO_2_ and He + CO_2_ are shown in Figure 11a. After CO_2_ hydrogenation at a steady state, the surface of the catalyst, especially TiO_2_, is expected to be saturated with CH_3_O* and HCOO*, and switching to CO_2_ allows the capturing of the activation process of CO_2_ by Re–H with less formate spillover. The MCR-resolved spectra obtained after a quasi-steady state reveal additional surface species at 1643, 1583, 1405, 1359, and 1307 cm^–1^ (Figure 11b, green) apart from κ^2^-HCOO* (Figure 11b, blue). Regarding κ^2^-HCOO*, H spillover from Re over TiO_2_ may create OH*, but the reaction D_2_ + CO_2_ (Figure 5b) suggested that CO_2_ was not activated over OH* into HCO_3_*, which could be further hydrogenated to HCOO*. These observations pointed out that the origin of HCOO* on TiO_2_ is via formate spillover from the Re site. The Ti^4+^ Lewis acidic sites at the interface could promote formate spillover similar to the surface chemistry observed over Ag on Al_2_O_3_ or ZrO_2_.^30,31^

On the other hand, the more complex spectra (Figure 11b, green) could be interpreted as H_2_O*, CO_3_*, and HCO_3_*^58,59^ or even HCOOCH_3_*.^40,41^ However, MCR-resolved spectra of CO_3_* and HCO_3_* identified under the CO_2_ hydrogenation condition over TiO_2_ did not match with the spectral features of the observed surface species excluding the possibility of (bi)carbonates. The bands at 1583, 1405, and 1359 cm^–1^ can be assigned to bridging formate (μ_2_-HCOO*) located over Re or the Re–O–Ti interface.^46^ The unassigned bands at 1643 and 1307 cm^–1^ could be carboxylate on rhenium (Re–COOH), 1647 cm^–1^,^60−62^ but this is unlikely with CO_2_ directly interacting with Re. On the other hand, CO_2_ can be hydrogenated molecularly over Re(CO)_3_ complexes (i.e., its hydride form).^63^ Thus-formed monodentate formate (κ^1^-HCOO*) binding on the rhenium center of Re(CO)_3_ complexes was reported around 1630 and 1280 cm^–1^,^61,62,64−66^ which is in close agreement with the observed bands. Moreover, those two bands were also observed via HCOOH adsorption over 1 wt% Re/TiO_2_ in a previous work (containing more single atoms).^19^ All assignments seem congruent with the results obtained during CO_2_ hydrogenation (Figure 4), in which these bands appeared after the formation of the Re(CO)_3_ complex. The complexity of the MCR-resolved spectrum suggested that CO_2_ activation over the Re-center and subsequent hydrogenation of CO_2_ over Re–H into μ_2_-HCOO*, located on Re or Re–O–Ti, and κ^1^-HCOO* are kinetically indistinguishable at the current time resolution as they appear in the same spectrum (Figure 11b, green).

The concentration profiles of the two kinetically distinguishable spectra obtained by MCR are shown in Figure 11c. During the CO_2_ phase, the amounts of Re–H and κ^1^-HCOO* decrease rapidly and gradually, respectively, while κ^2^-HCOO* increases via spillover of formate from Re to TiO_2_. This indicates that CO_2_ is immediately activated by Re–H and the formed formate spills over onto TiO_2_ very quickly. The gradual decrease of κ^1^-HCOO* indicates that the formate on Re is unstable under CO_2_ without hydrogen as expected, but the slow decay may imply that the formates on TiO_2_ may be reversibly transformed to κ^1^-HCOO* to some extent, thus delaying its decomposition. After switching to the H_2_ + CO_2_ phase, Re–H is rapidly regenerated and subsequently produces κ^1^-HCOO* (fast formate) as a source of CH_3_OH (Figure 11d). Interestingly, κ^2^-HCOO concentration decreases, indicating that the κ^2^-HCOO* close to the perimeter spills over back toward Re instantaneously. The proposed mechanisms of formate formation and spillover are summarized in Figure 11e.

The MCR-resolved spectra obtained in C–H stretching region (Figure S14) have shown a multi-band spectrum of HCOO* with a similar concentration profile of κ^1^-HCOO* in the C–O stretching region, while that of CH_3_O* on TiO_2_ is kinetically non-overlapping and behaves similarly to κ^2^-HCOO* during the CO_2_ phase but decays much slower in the H_2_ + CO_2_ phase. This CH_3_O* is produced via κ^1^-HCOO* hydrogenation to CH_3_OH that instantly adsorbed TiO_2_ to form relatively stable CH_3_O*.

**Figure 12:**
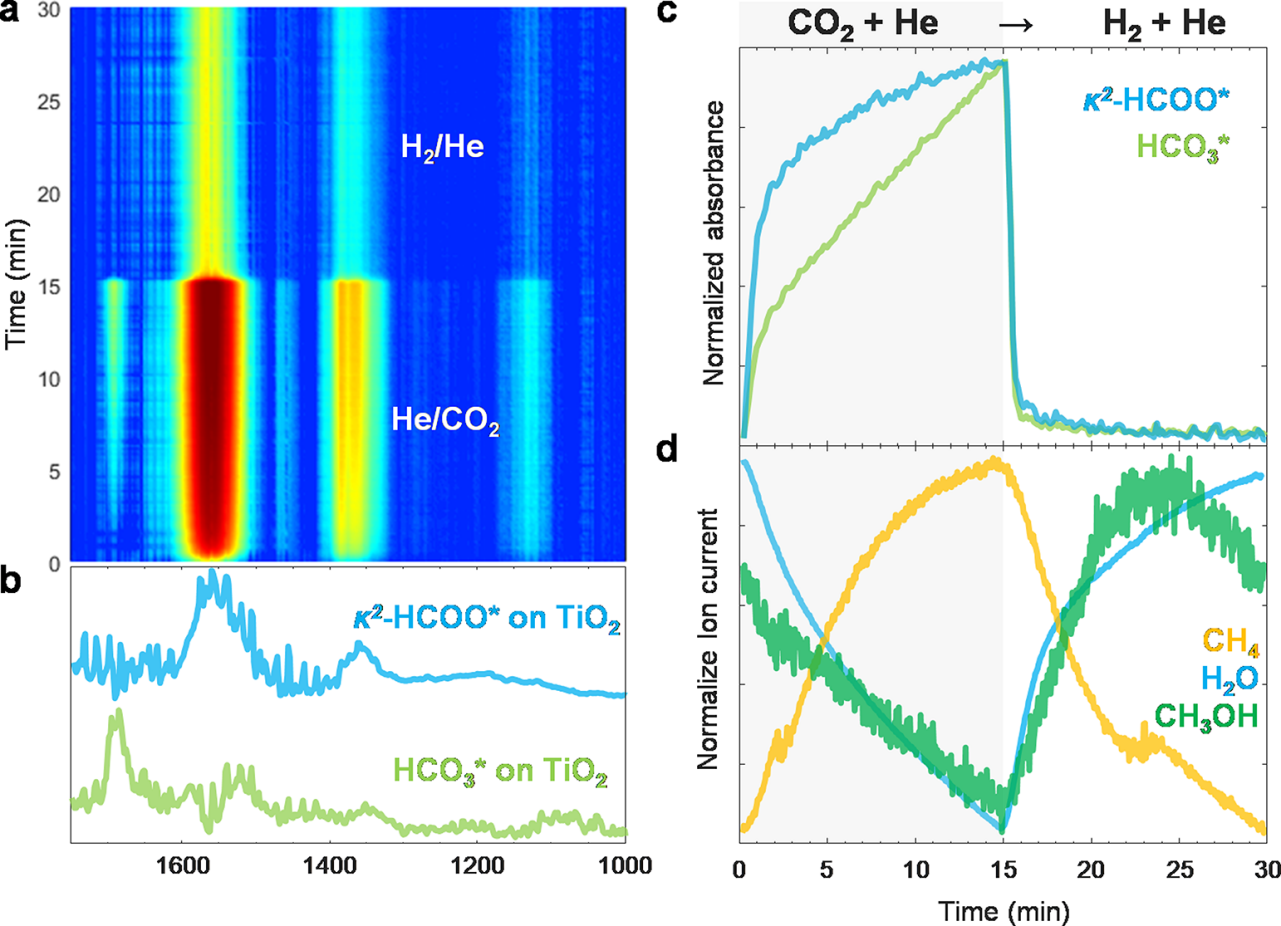
Transient DRIFTS study on CO_2_ hydrogenation over a 3 wt % Re/TiO_2_ catalyst. (a) Time-resolved DRIFT spectra upon transient concentration perturbation using H_2_ + He and He + CO_2_. (b) Components’ spectra obtained by multivariate spectral analysis applied on the time-resolved DRIFT spectra. (c) Concentration profiles of the corresponding components’ spectra obtained by the multivariate spectral analysis. (d) Corresponding normalized ion current signals obtained from MS. Reaction conditions: 10 mg catalyst, H_2_/He = He/CO_2_ = 3, *T* = 150 °C, *P* = 10 bar, *F*_total_ = 10 NmL min^–1^.

Furthermore, aiming to decouple the influences of two reactive components on surface species evolution, another type of transient experiment was performed by alternatingly passing (He^+^)CO_2_ and H_2_(+He) over Re/TiO_2_. From the pristine reduced catalyst under H_2_, surface species from CO_2_ started to evolve at every cycle after switching to CO_2_ until a quasi-steady state is reached, indicating the reaction between CO_2_ and adsorbed Re–H. Due to its transient nature, a short-lived species that cannot be detected under steady-state CO_2_ hydrogenation can be revealed (Figure 12a). The MCR resolved spectra show a characteristic band at 1690 cm^–1^ apart from κ^2^-HCOO* (Figure 12b), which can be identified as the formyl group (HCO*)^67−69^ or formic acid (HCOOH*).^42−44^ The missing corresponding C–H bonds suggested that such species could also be carboxylate (COOH*) on Re,^60−62^ or κ^1^- or κ^2^-HCO_3_* on TiO_2_.^59^ However, the formation of HCO_3_* was more congruent with the role of TiO_2_ support in CO_2_ activation to CO_3_* and HCO_3_* during CO_2_ hydrogenation in the absence of Re (Figure S7).

The concentration profile (Figure 12c) showed that κ^2^-HCOO* forms during the CO_2_ phase faster than HCO_3_* while both are rapidly converted off during the H_2_ phase. However, not all κ^2^-HCOO* was completely removed during the H_2_ phase (Figure 12a), confirming the existence of unreactive or slow formates as spectators. On the other hand, the vanishing of HCO_3_* suggested the short-lived nature that makes it unobservable during steady-state CO_2_ hydrogenation (Figure 4). HCO_3_* appeared only during the CO_2_ phase due to the limited hydrogenation (by Re–H or H*). Since CH_4_ is produced in combination with HCO_3_* formation during the CO_2_ phase (Figure 12d), hydrogenation of HCO_3_* into Re–CO could be the pathway for CH_4_ formation. This was supported by the observation of methyl (CH_3_*) on ReO_*x*_ at 2975 cm^–1^, which forms simultaneously during the CO_2_ phase (Figure S15).^70^ The SSITKA: D_2_ + CO_2_ to H_2_ + CO_2_ experiment (Figure S12) also confirms the hydrogenation of the CD_3_* intermediate into CHD_3_, which occurs before CH_4_ formation. A long decay in the CHD_3_ profile and delayed CH_4_ formation suggest that CD_3_* is less reactive than HCOO* intermediates forming CH_3_OH. Moreover, HCO_3_* hydrogenation to Re–CO is less favorable under common CO_2_ hydrogenation conditions due to the tendencies for HCOO* formation assisted by Re–H as well as the occupation of Re sites in the form of Re(CO)_3_. This is confirmed by the slower exchange between Re(CO)_3_ isotopes than HCOO* during the SSITKA: ^13^CO_2_ + H_2_ to ^12^CO_2_ + H_2_ experiment (Figure 6). All mechanistic insights from transient experiments affirm the main intermediates for both CH_3_OH and CH_4_ formation over sub-nano cluster Re/TiO_2_.

Unfortunately, the insight into intermediates of HCOOCH_3_ remains missing in this study due to the pressure limitation. The huge pressure gaps among 10, 56, and 331 bars are significant in terms of HCOOCH_3_ selectivity. However, the slightly higher CH_3_OH selectivity seems to be due to the suppression of HCOO* and/or HCOOCH_3_ decomposition toward CO at a higher pressure. The mechanism can be extrapolated from our previous works in which CH_3_OH reacts with HCOOH retained by HCOO* spillover^30,31^ and surface-coverage-dependent productivity (Figure 1). HCOOH formation from HCOO* was proposed over cluster-sized Cd_4_/TiO_2_ via the hydride-coordinated metal center and proton-bonded O site of TiO_2_,^71^ which could be similar in the case of Re/TiO_2_.

### 2.7 How Does Bifunctionality of Re Supported on TiO_2_ Drive Methanol Formation?

**Figure 13:**
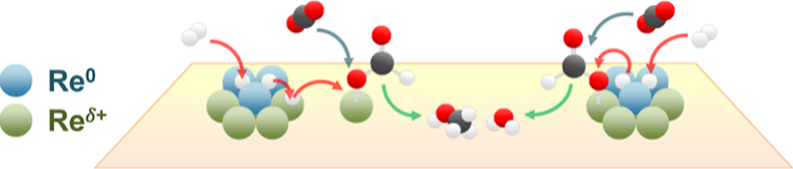
Simplified mechanism involving Re^0^ and Re^δ+^ species.

The mechanistic insights uncover the origin of high performance observed for the Re/TiO_2_ catalyst at low temperatures. For 3 wt % Re/TiO_2_, redispersion of Re clusters into smaller clusters and single-atom Re improved the CH_3_OH formation performance. The redispersion of Re clusters resulted in an increase in the number of highly dispersed Re^δ+^ species while maintaining a large number of Re^0^ species in the (sub)nano-clusters. The Re^0^ sites play a role as an H_2_ activator and transfer H to the Re^δ+^ species present near the perimeter or rather single-atom Re^δ+^. The latter forms an Re(CO_3_) complex and its hydride (Re–H). Such Re^δ+^ species can activate CO_2_ molecularly, especially after κ^1^-HCOO* formation. Notably, the observed κ^1^-HCOO* is rarely reported over metal-supported catalysts since its formation is likely not possible over the extended metal surface (e.g., Re^0^), yet they can be identified over the metal active center of molecular complexes (e.g., Re, Ru, and Ir complexes).^72−74^ Further hydrogenation of κ^1^-HCOO* to CH_3_OH requires H supplied from Re^0^ species, thus requiring two types of sites for efficient hydrogenation of κ^1^-HCOO*. The simplified mechanism is shown in Figure 13.

The mechanistic propositions given above are congruent with the structure relationship reported previously.^19^ In the previous work, the wt % of Re/TiO_2_ was studied at 0.2, 1, 5, 10, and 20 wt %. The highest CH_3_OH selectivity was obtained from the 1 wt % Re/TiO_2_, which contained the subnanometer size of the Re species. The CH_4_ selectivity gradually increases with the loading of at 5–20 wt %, which should be related to the increased fraction of Re^0^ species that can over-hydrogenate CO* to CH_4_. However, the isolated single Re species that is more dominantly present for the 0.2 wt % Re sample favors CO formation via formate decomposition since single atom Re^δ+^ species alone lack the ability to activate H_2_ when formate is coordinating to the Re center and further hydrogenate κ^1^-HCOO* to CH_3_OH. Therefore, it can be concluded that Re/TiO_2_ requires both Re^0^ and Re^δ+^ species for efficient methanol formation. Insufficient H supply from Re^0^ species can lead to formate decomposition to CO, while the excess leads to over-hydrogenation to CH_4_. Re^δ+^ species can activate CO_2_ and stabilize κ^1^-HCOO* for faster hydrogenation to CH_3_OH.

## 3 Conclusions

In summary, Re/TiO_2_ was superior to Cu/ZnO/Al_2_O_3_ for CO_2_-to-CH_3_OH at low temperatures thanks to the distinct reaction pathway, activated by the unique interactions between Re and TiO_2_ and less formation of HCOOCH_3_ over Lewis acid sites on Al_2_O_3_. During operation using 3 wt % Re/TiO_2_, Re clusters become more dispersed, increasing the number of cationic Re single atoms and forming rhenium tricarbonyl complexes. On the other hand, metallic Re species remain a major fraction and act as an H_2_ activator for H-spillover to the cationic Re sites. The formation of Re–H is the first step in CO_2_ activation to monodentate formate over the cationic Re species before its further hydrogenation to CH_3_OH or spillover onto TiO_2_ support, becoming less reactive bidentate formate (spectator). The formed CH_3_OH tends to adsorb strongly over the hydroxyl sites on TiO_2_, forming methoxy species (spectator). Surface bicarbonates are observed as an intermediate for carbonyl species, which is further hydrogenated into methyl species, leading to CH_4_ formation. These mechanistic insights explain the unique reactivity of Re/TiO_2_ with two kinds of sites, being able to form active formate and further hydrogenation at low temperatures and can help polish the concept to design more selective low-temperature methanol synthesis catalysts.

## 4 Experimental Section

Experimental procedures, commercial chemicals, catalyst preparation procedures, instrument specifications, and characterization techniques are covered in greater detail in the Supporting Information.

## Supporting Information Available

Assignment of surface species; *m*/*z* ratio obtained using a mass spectrometer; effects of temperatures on the catalytic activity of Re/TiO_2_; catalytic stability of Re/TiO_2_; elemental mapping of Re/TiO_2_; *in situ* XANES and Raman of Re/TiO_2_; *in situ* DRIFTS during D_2_ + CO_2_ reaction over Re/TiO_2_; *in situ* DRIFTS during H_2_ + ^13^CO_2_ reaction over Re/TiO_2_; *in situ* DRIFTS and MCR during H_2_ + CO_2_ reaction over TiO_2_; *in situ* DRIFTS during CH_3_OH adsorption and reaction with D_2_ over Re/TiO_2_; *in situ* DRIFTS during CH_3_OH adsorption and reaction with D_2_ over TiO_2_; *in situ* DRIFTS during H_2_ + CO reaction over Re/TiO_2_; *in situ* DRIFTS-MS and MCR during SSITKA CO_2_ + H_2_ to CO_2_ + D_2_; *in situ* DRIFTS-MS and MCR during SSITKA CO_2_ + H_2_ to CO_2_ + D_2_ to CO_2_ + H_2_; *in situ* Raman of Re/TiO_2_ during transient H_2_ + CO_2_ vs He + CO_2_; *in situ* DRIFTS and MCR during transient H_2_ + CO_2_ and H_2_ + He; and *in situ* DRIFTS and MCR during transient H_2_ + He and He + CO_2_ (PDF)
