# Supplementary material for: Bifunctionality of Re Supported on TiO2 in Driving Methanol Formation in Low-Temperature CO2 Hydrogenation
Source: ACS Catal. 2023 Aug 1;13(16):10734–50. doi: 10.1021/acscatal.3c01599 (PMC10442859; doi:10.1021/acscatal.3c01599)
Supplement: Supplementary file 1 — cs3c01599_si_001.pdf [file cs3c01599_si_001.pdf]

Supporting Information

Bifunctionality of Re supported on TiO_2 in driving methanol formation in low-temperature CO_2 hydrogenation

Nat Phongprueksathat,† Kah Wei Ting,‡ Shinya Mine,‡ Yuan Jing,‡ Ryo Toyoshima,§ Hiroshi Kondoh,§ Ken-ichi Shimizu,‡ Takashi Toyao,*, ‡ Atsushi Urakawa,*, †

† Catalysis Engineering, Department of Chemical Engineering, Delft University of Technology, Van der Maasweg 9, 2629 HZ Delft, Netherlands

‡ Institute for Catalysis, Hokkaido University, N-21, W-10, Sapporo 001-0021, Japan.

§ Department of Chemistry, Keio University, 3-14-1 Hiyoshi, Kohoku-ku, Yokohama 223-8522, Japan

*E-mail: toyao@cat.hokudai.ac.jp (T.T.).

*E-mail: A.Urakawa@tudelft.nl (A.U.).

Contents

1. Supplementary experimental 3

1.1. Materials and catalyst preparation 3

1.2. Catalyst characterization procedure 3

1.3. Catalytic activity testing procedure at high pressure 3

1.4. Transient experimental setup and procedures of *operando* XAS, Raman, and DRIFTS 4

1.4.1. *Operando* XAS 4

1.4.2. *Operando* Raman 4

1.4.3. *Operando* DRIFTS and SSITKA 4

1.4.4. Multivariate spectral analysis 5

1.4.5. AP-XPS 5

2. Supplementary results 8

2.1. Catalytic activity at industrially-relevant conditions (56 bars) 8

2.2. Elemental mapping 9

2.3. *In situ* characterization of the catalyst during CO_2 hydrogenation 9

2.4. DRIFTS: CO_2 hydrogenation (using D_2) over Re/TiO_2 10

2.5. DRIFTS: $^{13}CO_2$ hydrogenation over Re/TiO_2 11

2.6. DRIFTS: CO_2 hydrogenation over TiO_2 12

2.7. DRIFTS: CH_3OH adsorption over Re/TiO_2 and titration of CH_3O^* adlayer with D_2 13

2.8. DRIFTS: CH_3OH adsorption over TiO_2 and titration of CH_3O^* adlayer with D_2 14

2.9. DRIFTS: CO hydrogenation over Re/TiO_2 15

2.10. DRIFTS-SSITKA: H_2+CO_2 → D_2+CO_2 16

2.11. DRIFTS-SSITKA: H_2+CO_2 → D_2+CO_2 → H_2+CO_2 17

2.12. Raman: Transient H_2+CO_2 vs He+CO_2 18

2.13. DRIFTS: Transient H_2+CO_2 vs He+CO_2 19

2.14. DRIFTS: Transient H_2+He vs He+CO_2 20

References 21

1. Supplementary experimental

1.1. Materials and catalyst preparation

The obtained reagents were used as received. TiO_2 (ST-01) was purchased from Ishihara Sangyo Co., Ltd. Its BET (Brunauer−Emmett−Teller) specific surface area is 188 m^2/g^{-1}. Re_2O_7 and ReO_2 were purchased from Strem Chemicals Inc. and Hydrus Chemical Inc., respectively. NH_4ReO_4 and metallic Re were purchased from Sigma Aldrich. The commercial methanol synthesis catalyst ($Cu/ZnO/Al_2O_3$) was purchased from Alfa Aesar (Product ID: 45776).

Precursors for Re/TiO_2 were prepared by mixing the support material with the metal sources, that is, an aqueous solution of NH_4ReO_4, For the preparation of Re/TiO_2, typically 0.072 g of NH_4ReO_4 was added to a glass vessel (500 mL) containing 100 mL of deionized water ([Re] = 0.0027 M). After sonication (1 min)to completely dissolve the NH_4ReO_4, TiO_2 (4.95 g) was added to the solution. The mixed solution was then stirred at 200 rpm for 30 min at room temperature. Subsequently, the solvent of the mixture was evaporated at T = 50 °C, followed by drying in the air (T = 110 °C; t = 12 h). The thus obtained material was calcined (T= 500 °C, t = 3 h, in the air).

1.2. Catalyst characterization procedure

Scanning electron microscope (SEM) images were obtained by Hitachi HD-2000. High-angle annular dark-field imaging (HAADF) was performed using a JEM-ARM200F scanning transmission electron microscope (STEM). Samples were prepared by dropping an ethanol solution containing the catalyst on carbon-supported Cu grids.

1.3. Catalytic activity testing procedure at high pressure

The catalytic tests were carried out in a high-pressure setup as reported elsewhere.[1] In a typical test, 500 mg catalyst was packed between quartz wool inside a 1/4 inch fixed-bed continuous flow reactor (ID 2.79 mm). The catalyst was reduced in situ at 450 °C with 90% H_2/Ar (25 NmL min^{-1}) for 1 h under atmospheric pressure. After cooling down to 30 °C, the H_2/CO_2/Ar mixture with vol% of 69%/23%/8% was fed into the reactor and pressurized to 360 bar (the reactant pressure is 331 bar). The total flow rate of the gas mixture is kept at 16.7 NmL min^{-1} to achieve a gas-hourly space velocity of 2000 hr^{-1} equivalents. The products were analyzed by an online gas chromatograph (Bruker, GC-450) equipped with a flame ionization detector for methanol, methyl formate, diethyl ether, and other hydrocarbons, and a thermal conductivity detector for permanent gases e.g. CO_2, H_2, Ar, CO, CH_4.

1.4. Transient experimental setup and procedures of *operando* XAS, Raman, and DRIFTS

The flow of gases (H_2, CO_2, and He) is controlled by 6 mass flow controllers (Bronkhorst). Switching between two reactant gas streams is done by a 4-way valve. The pressure of the two gas streams (to the cell and vent) is controlled by back pressure regulators (Bronkhorst). The outlet gas stream is analyzed by a Pfeiffer OmniStar GSD 300C mass spectrometer.

Before the measurements, the sample is reduced *in situ* at 500 °C in the H_2 stream (20 NmL min^{-1} H_2) for 1 h and subsequently cooled to a reaction temperature of 150 °C in the He stream. The cell is pressurized to 10-20 bar and immediately exposed to the reactant mixture (CO_2:H_2 = 1:3 molar ratio, total flow 20 NmL min^{-1}) at the same pressure by the switching valve. The transient experiment utilizes a periodic perturbation of a system by external parameters (stimulation) to influence the concentration of active species.[2] This experiment is performed in the above-mentioned setup by using a switching valve to change the stream of reactant gases to introduce the periodic concentration perturbation.

1.4.1. *Operando* XAS

Operando XAS measurements were carried out using a fixed-bed capillary reactor (ID: 2 mm) at 150 °C, 10 bar, and H_2:CO_2 = 3:1 coupled with detection of the formed products by the mass spectrometer. XAS measurements were performed over Re(3 wt%)/TiO_2 at Re L_3-edge (10.54 keV) will be performed.

1.4.2. *Operando* Raman

Raman measurements were performed using a BWTEK dispersive i-Raman portable spectrometer equipped with a 785 nm excitation laser and a TE-cooled linear array detector. The reaction was carried out in a fixed-bed capillary reactor (ID: 2 mm) with identical procedures to *operando* XAS.

1.4.3. *Operando* DRIFTS and SSITKA

The catalyst powder (10-15 mg) is located in a cylindrical cavity (3 mm in diameter and 3 mm vertical length) of a custom-made high-pressure reaction cell (tested up to 40 bar). The cell is mounted in a Harrick Praying Mantis diffuse reflection (DRIFTS) accessory. The spectra were collected using a Thermo Scientific Nicolet 6700 FT-IR spectrometer equipped with a liquid-nitrogen-cooled MCT detector at 4 cm^{-1} resolution. The spectra were acquired continuously every 10 seconds in a time-resolved manner to monitor the reaction, stabilization process of the catalysts as well as the evolution of surface species. No baseline correction was applied to the time-resolved spectra due to the baseline movement.

1.4.4. Multivariate spectral analysis

Multivariate spectral analysis is performed by the Multivariate Curve Resolution-Alternating Least Squares (MCR-ALS) algorithm, as described elsewhere.[3,4] MCR is a chemometric method used for better data processing and deconvolution of the complex spectra down to individual components based on kinetic resolution. It can deliver the pure response profiles (e.g. spectra, pH profiles, time profiles, elution profiles) of the chemical species of an unresolved mixture when no previous information is available about the nature and composition of these mixtures.

1.4.5. AP-XPS

Ambient pressure X-ray photoelectron spectroscopy (AP-XPS) measurements were performed at beam line13B of Photon Factory (PF) at the High Energy Accelerator Research Organization (KEK). A powder of Re/TiO_2 with Re loading of 3 wt%, pre-reduced under H_2 at 500 °C for 30 min, were coated on a Si substrate by using deionized water as a dispersant with a drop-and-dry method. The temperature of samples was measured by using a thermocouple directly attached to the sample holder in the analysis chamber. The gases were introduced into the chamber by using variable leak valves. The samples were pretreated by exposure to H_2 (0.1 Torr) at 450 °C for 30 min followed by cooling to 150 °C under the H_2 atomosphere. Gases were then introduced into analysis chamber and the all the XPS spectra were collected at 150 °C. Re 4f measurements were performed with a photon energy of 630 eV. Binding energy was calibrated using the Ti $2p_{3/2}$ peak of Ti^{4+} species (TiO_2; 485.5 eV). XPS spectra were analyzed with convolution of Gaussian and Lorentzian with a Shirley background in the range of 34-46.5 eV. An asymmetric Doniach–Sunjic peak shape was used to fit the peaks for metallic rhenium.

Tables S1. Assignement of surface species

Wavenumber (cm^{-1})			Vibrational mode	Band assignment	Ref.
**$CO_2 + H_2$ Feed**	**$CO_2 + D_2$ Feed**	**$^{13}CO_2 + H_2$ Feed**			
3716	2704	3716	ν(O–H)	Hydroxy (OH*) on TiO_2	5
2950		2941	ν_{as}(C–O) + δ(C–H)	Formate (HCOO*) on TiO_2	6-8
2927		2918	ν_{as}(C–H)	Methoxy (CH_3O*) on TiO_2	7,9,10
2869	2121	2860	ν(C–H)	Formate (HCOO*) on TiO_2	6-8
2829	2065	2821	ν_s (C–H)	Methoxy (CH_3O*) on TiO_2	7,9,10
1960	1335	1960	ν(Re–H)	Rhenium hydride (Re–H)	11-15
1656			ν_{as}(C–O)	Monodentate bicarbonates (HCO_3*) on TiO_2	16
1618			ν_{as}(C–O)	Bidentate bicarbonates (b-HCO_3*) on TiO_2	16
1583			ν_{as}(C–O)	Monodentate carbonate (m-CO_3*) on TiO_2	16
1560	1560	1527	ν_{as}(C–O)	Formate (HCOO*) on TiO_2	6-8
1503			ν_{as}(C–O)	Bidentate bicarbonates (b-HCO_3*) on TiO_2	16
1429			ν_{as}(C–O)	Monodentate bicarbonates (HCO_3*) on TiO_2	16
1369			ν_{as}(C–O)	Monodentate carbonate (CO_3*) on TiO_2	16
1357	1357	1334	ν_s(C–O)	Formate (HCOO*) on TiO_2	6-8
1334			ν_s(C–O)	Adsorbed carbon dioxide (CO_2*)	16
1223			ν_b(C–O–H)	Bidentate bicarbonates (HCO_3*) on TiO_2	16

Tables S2. Mass to charge ratio mass spectrometer

**Gases**	**m/z**
H_2	2
He	4
CH_4	15
$^{13}CH_4$	17
CH_3D	17
H_2O	18
CH_2D_2	18
CHD_3	19
CD_4	20
CO	28
^{13}CO	29
CH_3OH	31
$^{13}CH_3OH$	33
CH_3OD	33
CD_3OH	35
CD_3OD	36
CO_2	44
$^{13}CO_2$	45

2. Supplementary results

2.1. Catalytic activity at industrially-relevant conditions (56 bars)

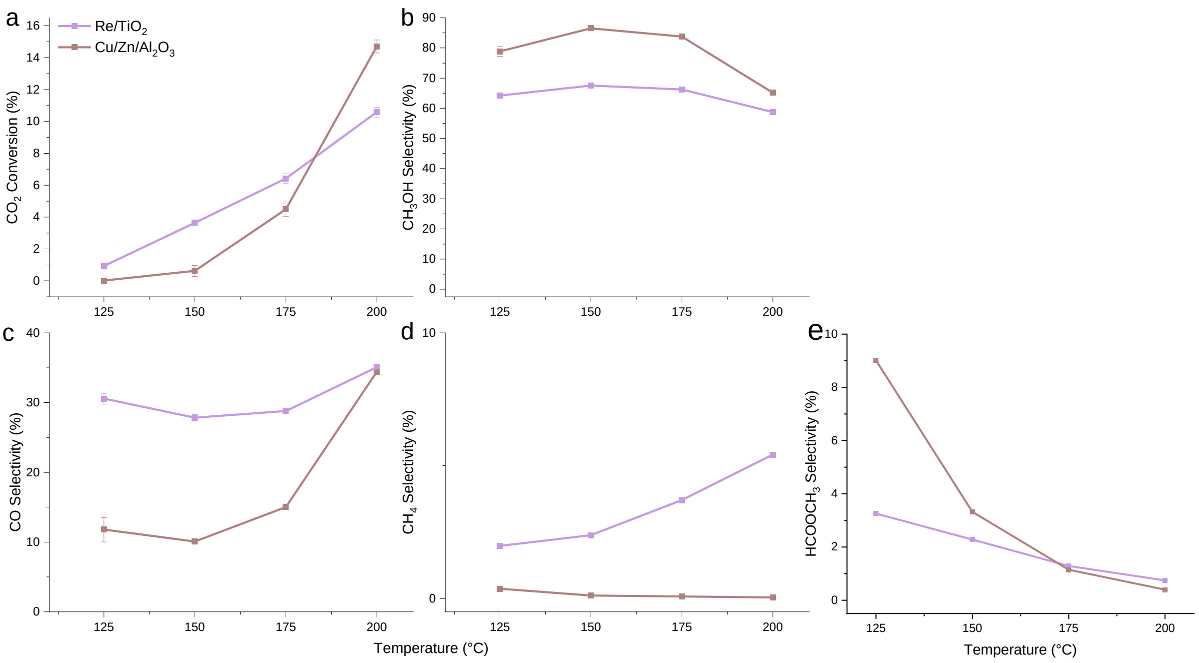

Figure S1 Effects of temperatures on **a** CO_2 conversion and product selectivity of **b** CH_3OH **c** CO and **d** CH_4 **e** $HCOOCH_3$ during CO_2 hydrogenation over 3 wt% Re/TiO_2 and $Cu/ZnO/Al_2O_3$. Reaction conditions: T = 125-200 °C, P = 56 bar, SV = 3.5 NL g_{cat}^{-1} h^{-1}, H_2/CO_2 = 3.

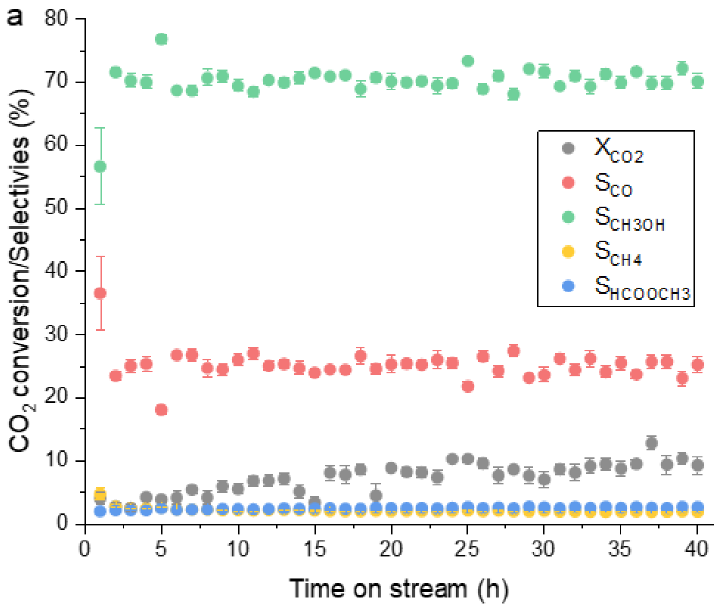

Figure S2 Catalytic stability during CO_2 hydrogenation of a Re/TiO_2.
Reaction conditions: T = 150 °C, P = 56 bar, H_2/CO_2 = 3, SV = 3505 NmL g_{cat}^{-1} h^{-1}.

2.2. Elemental mapping

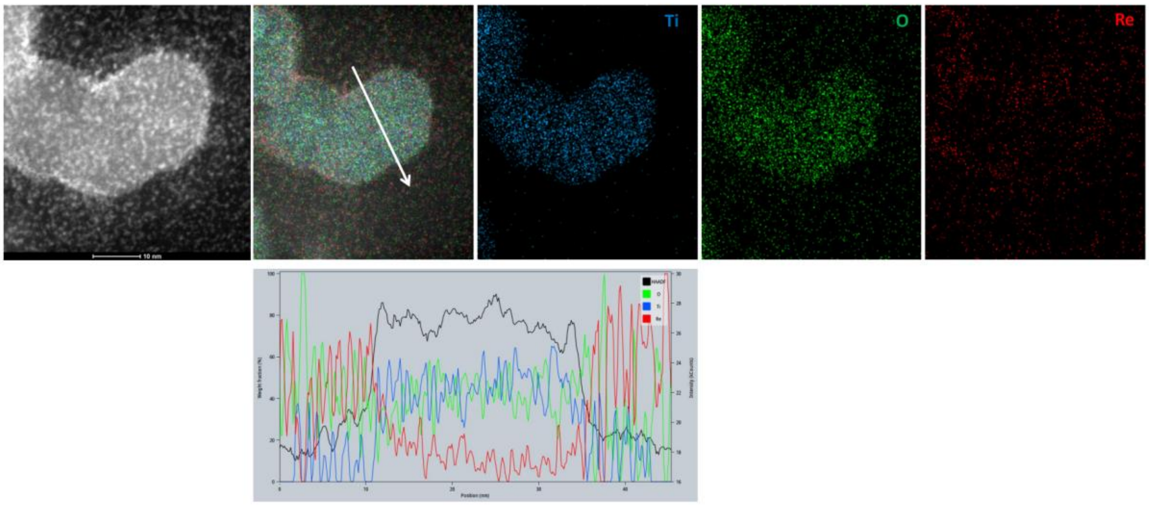

Figure S3 Elemental mapping of fresh 3 wt% Re/TiO_2.

2.3. *In situ* characterization of the catalyst during CO_2 hydrogenation

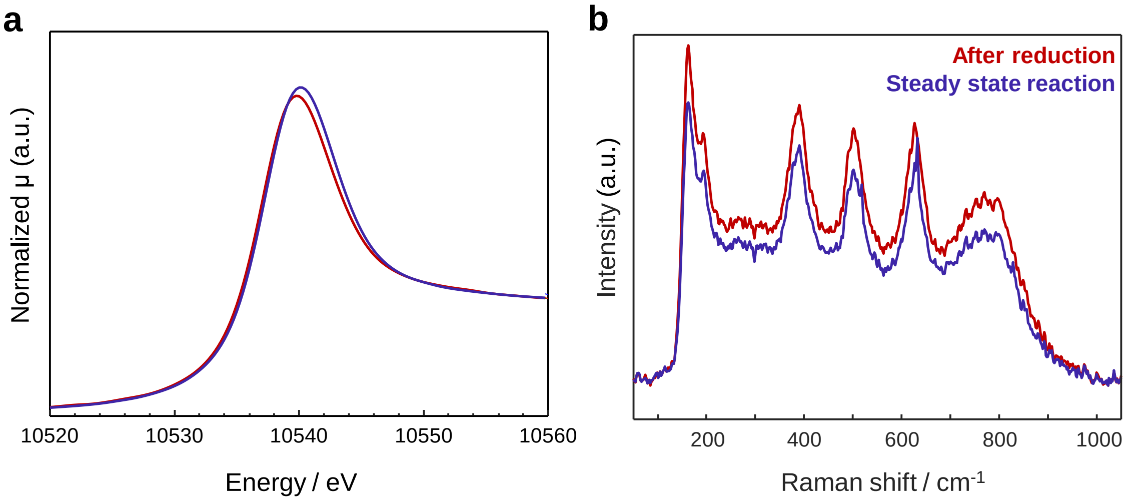

Figure S4 *In situ* characterization of 3 wt% Re/TiO_2 catalyst after reduction and during steady state reaction with H_2+CO_2: a *operando* Re L_3-edge XANES spectra and b *operando* Raman spectra. Reaction conditions: ca. 10 mg catalyst, H_2/CO_2 = 3, T = 150 °C, P = 10 bar, F_{total} = 10 NmL min^{-1}.

2.4. DRIFTS: CO_2 hydrogenation (using D_2) over Re/TiO_2

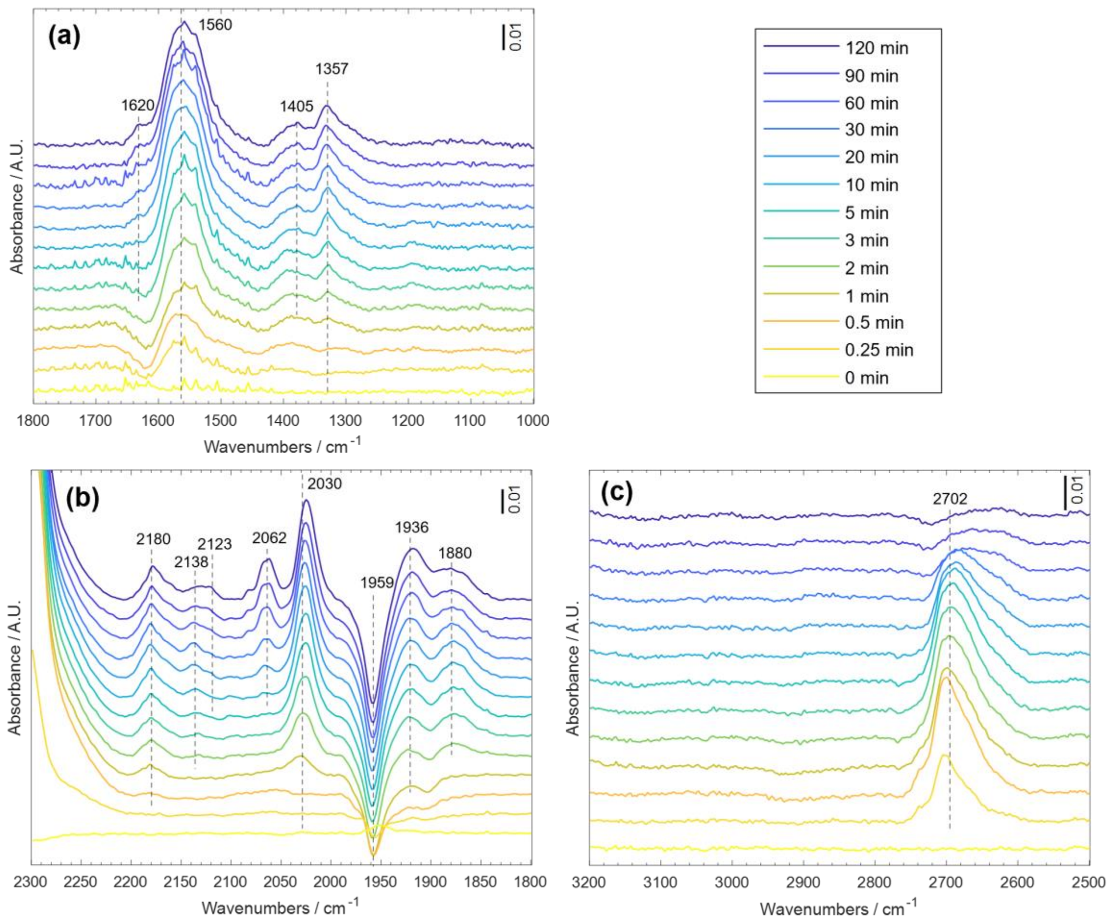

Figure S5 Temporal evolution of surface species obtained from *in situ* DRIFTS during reaction with D_2+CO_2 over 3 wt% Re/TiO_2. Reaction conditions: 10 mg catalyst, H_2/CO = 3, T = 150 °C, P = 10 bar, F_{total} = 10 NmL min^{-1}.

2.5. DRIFTS: $^{13}CO_2$ hydrogenation over Re/TiO_2

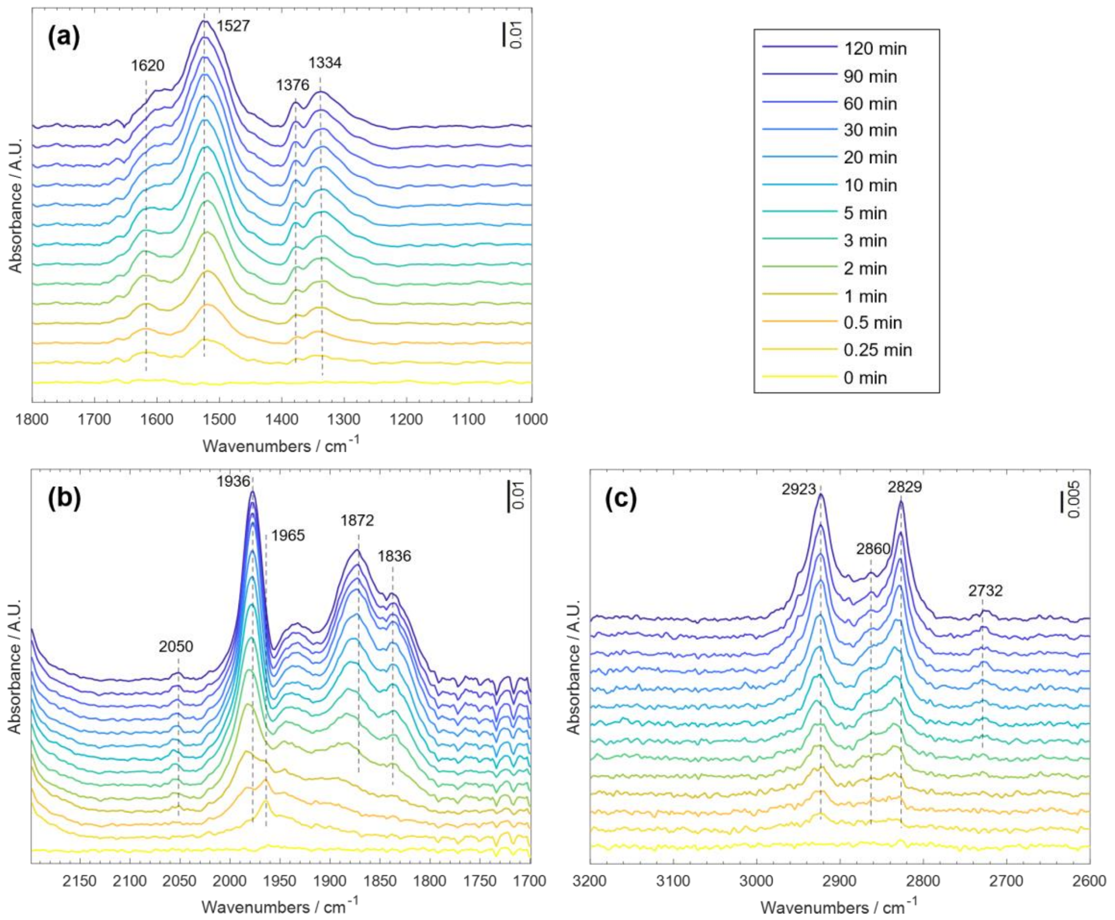

Figure S6 Temporal evolution of surface species obtained from *in situ* DRIFTS during reaction with H_2+$^{13}CO_2$ over 3 wt% Re/TiO_2. Reaction conditions: 10 mg catalyst, H_2/CO_2 = 3, T = 150 °C, P = 10 bar, F_{total} = 10 NmL min^{-1}.

2.6. DRIFTS: CO_2 hydrogenation over TiO_2

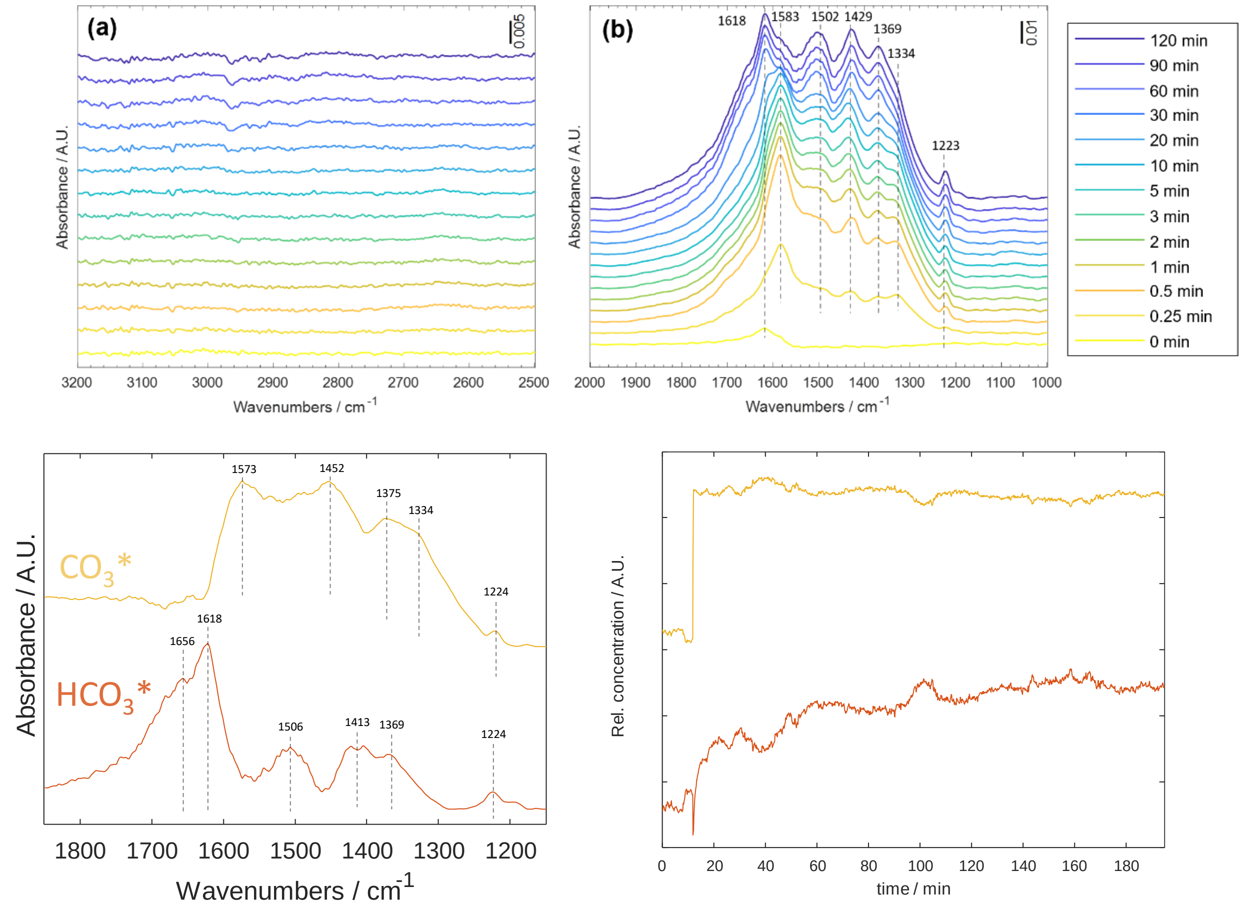

Figure S7 Temporal evolution of surface species obtained from *in situ* DRIFTS during reaction with H_2+CO_2 over TiO_2 (top), components spectra obtained by MCR applied on the time-resolved DRIFT spectra (bottom-left), and concentration profiles of the spectra of the corresponding components obtained by MCR (bottom-right). Reaction conditions: 10 mg catalyst, H_2/CO_2 = 3, T = 150 °C, P = 10 bar, F_{total} = 10 NmL min^{-1}.

2.7. DRIFTS: CH_3OH adsorption over Re/TiO_2 and titration of CH_3O^* adlayer with D_2

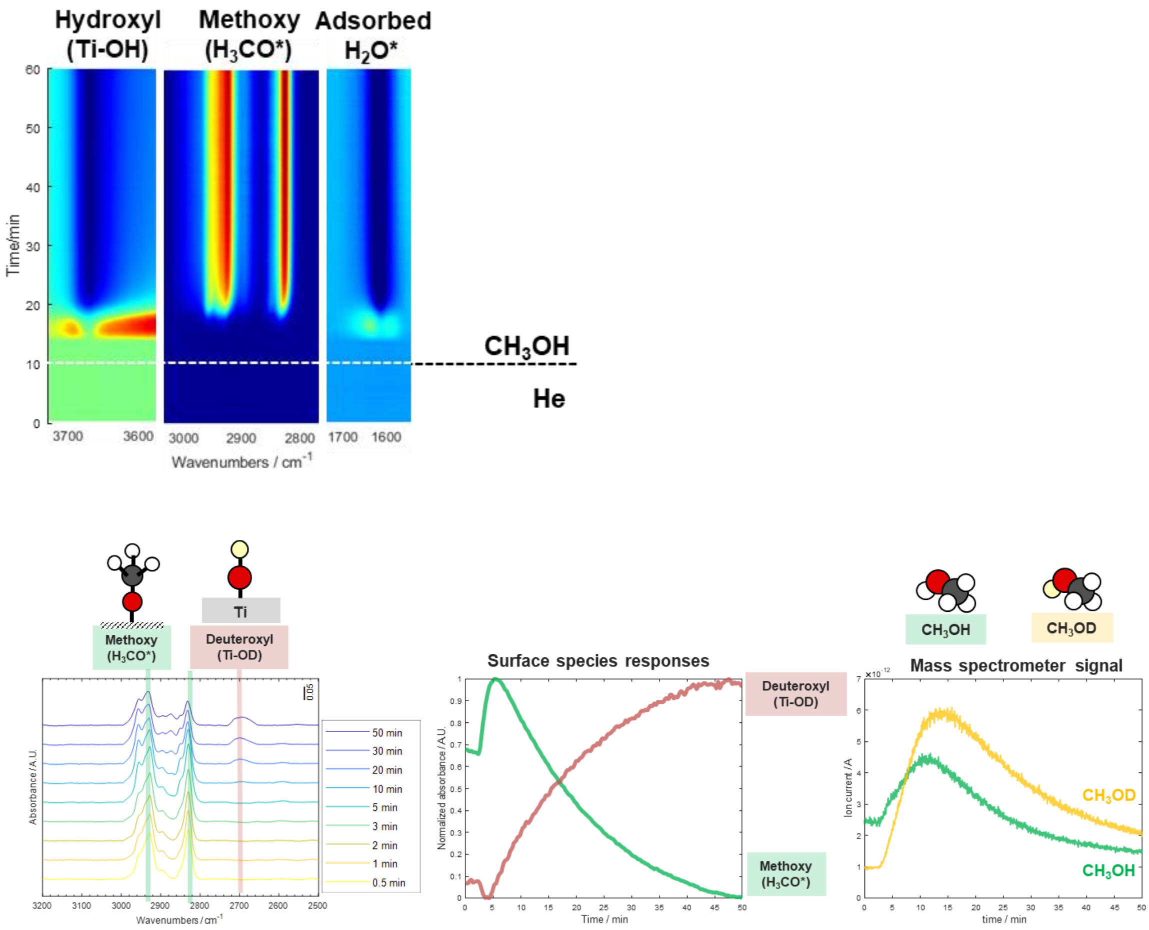

Figure S8 Time-resolved CH_3OH adsorption over Re/TiO_2 (upper) and the reaction of CH_3O^* adlayer with D_2 after flushing with He (bottom). Reduction condition: 300 ºC under 10 NmL min^{-1} of H_2 for 1 h. (Pre-reduced at 500 °C with H_2 and passivated with $1\%O_2/N_2$). Reaction conditions: ca. 10 mg catalyst, 2000 ppm CH_3OH in N_2, T = 150 °C, P = 5 bar, F_{total} = 20 NmL min^{-1}.

2.8. DRIFTS: CH_3OH adsorption over TiO_2 and titration of CH_3O^* adlayer with D_2

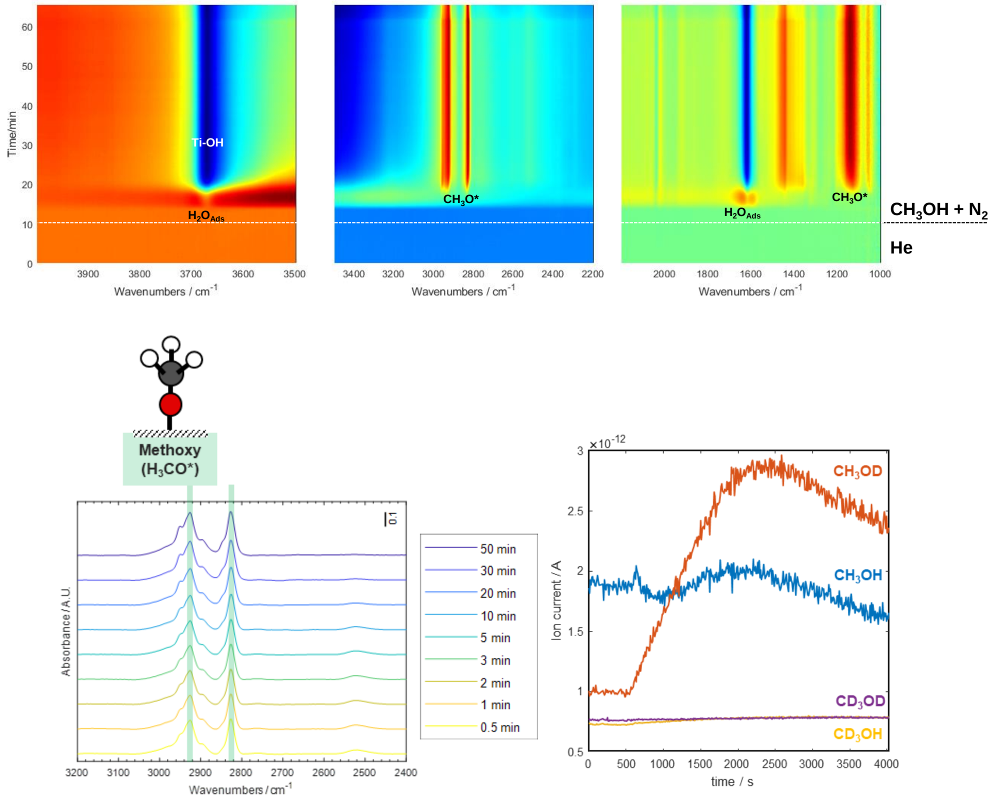

Figure S9 Time-resolved CH_3OH adsorption over TiO_2 (upper) and the reaction of CH_3O^* adlayer with D_2 after flushing with He (bottom). Reduction condition: 300 ºC under 10 NmL min^{-1} of H_2 for 1 h. Reaction conditions: ca. 10 mg catalyst, 2000 ppm CH_3OH in N_2, T = 150 °C, P = 5 bar, F_{total} = 20 NmL min^{-1}.

2.9. DRIFTS: CO hydrogenation over Re/TiO_2

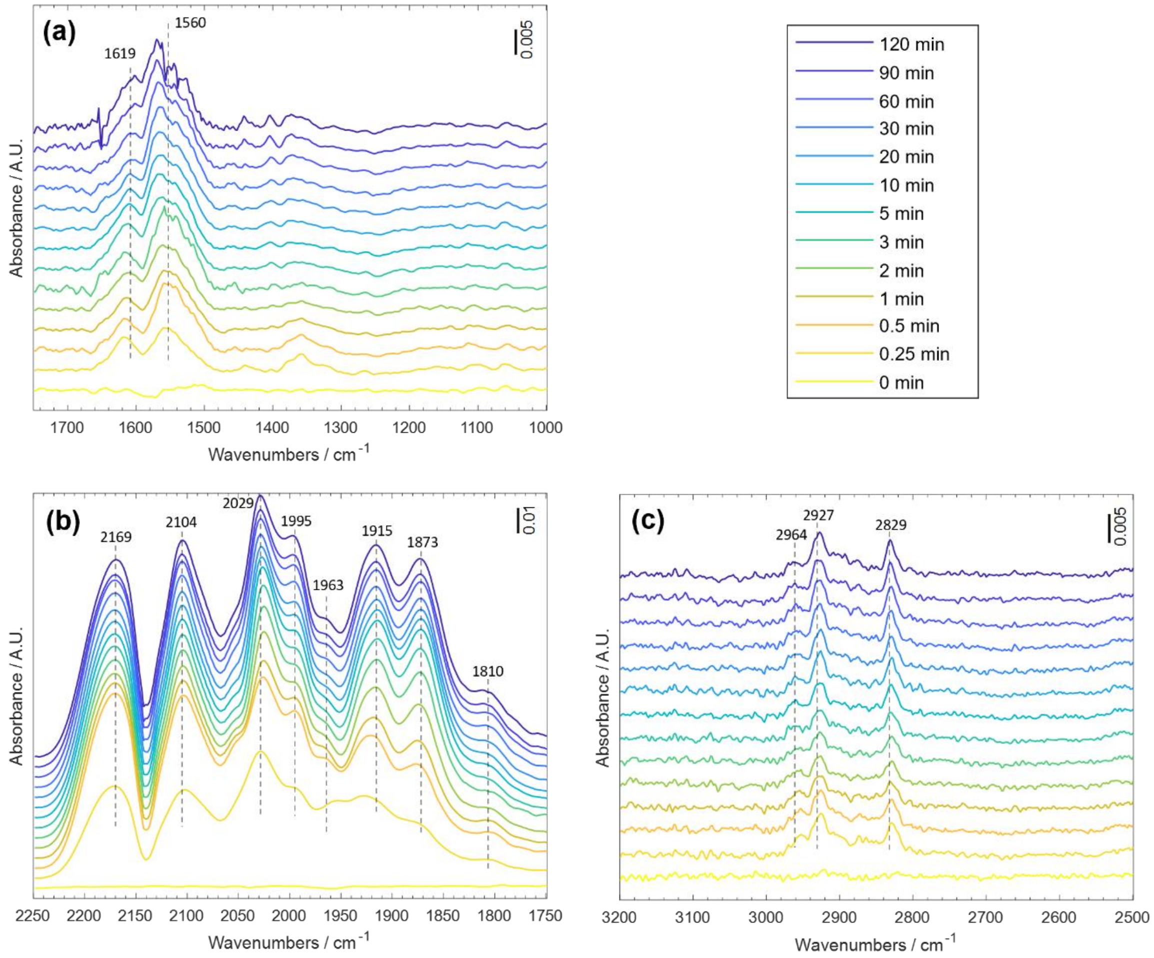

Figure S10 Temporal evolution of surface species obtained from *in situ* DRIFTS during reaction with H_2+CO over 3 wt% Re/TiO_2.Reduction condition: 300 ºC under 10 NmL min^{-1} of H_2 for 1 h. (Pre-reduced at 500 °C with H_2 and passivated with 1%O_2/N_2). Reaction conditions: 10 mg catalyst, H_2/CO = 3, T = 150 °C, P = 10 bar, F_{total} = 10 NmL min^{-1}.

2.10. DRIFTS-SSITKA: $H_2+CO_2 \rightarrow D_2+CO_2$

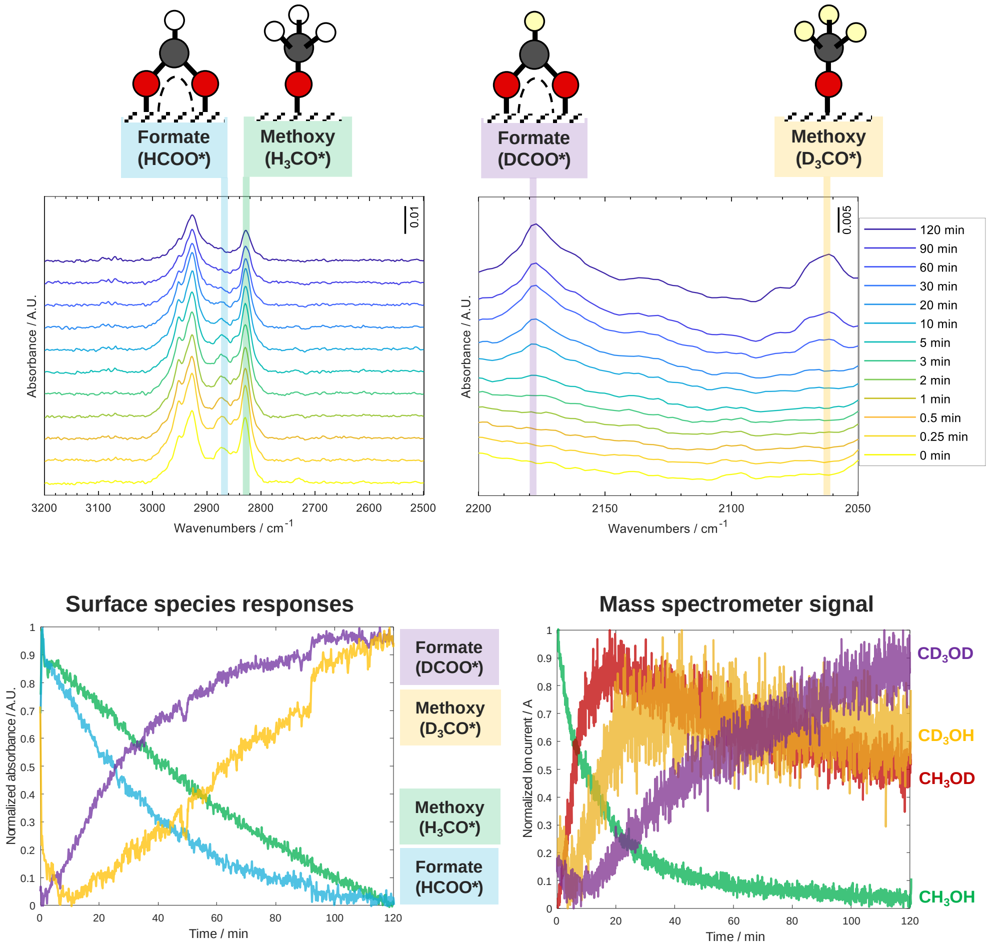

Figure S11 Transient responses of surface species and gas products during the steady-state isotopic switching from CO_2+H_2 to CO_2+D_2. Time-resolved DRIFT spectra of a DCOO* and CD_3O*, and b HCOO* and CH_3O* (top) Concentration profiles of the spectra of the corresponding components obtained by MCR (bottom-left). Corresponding normalized ion current signal of isotope-labeled products bottom-right). Reaction conditions: 10 mg catalyst, H_2(or D_2)/CO_2 = 3, T = 150 °C, P = 10 bar, F_{total} = 10 NmL min^{-1}.

2.11. DRIFTS-SSITKA: $H_2+CO_2 \rightarrow D_2+CO_2 \rightarrow H_2+CO_2$

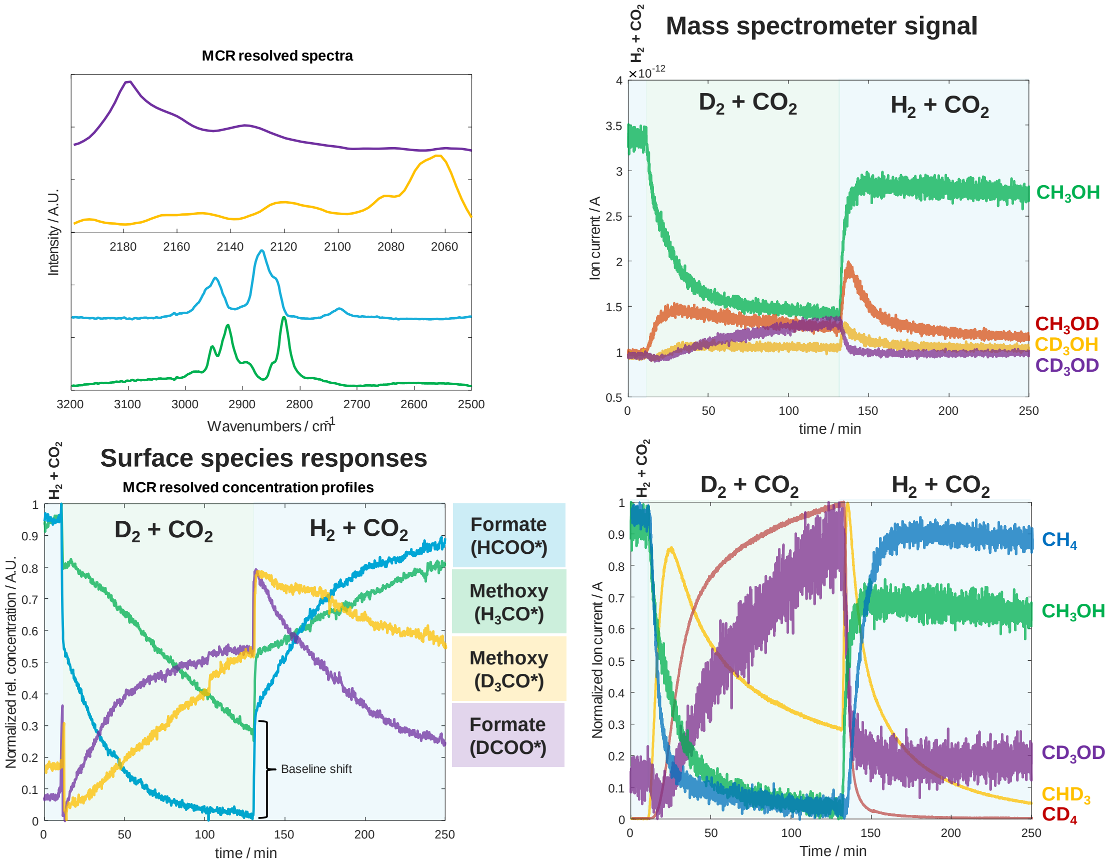

Figure S12 Transient responses of surface species and gas products during the steady-state isotopic switching from CO_2+H_2 to CO_2+D_2 to CO_2+H_2. Components spectra obtained by MCR applied on the time-resolved DRIFT spectra (top-left). Concentration profiles of the spectra of the corresponding components obtained by MCR (bottom-left). Corresponding normalized ion current signal of isotope-labeled products (right). Reaction conditions: 10 mg catalyst, H_2(or D_2)/CO_2 = 3, T = 150 °C, P = 10 bar, F_{total} = 10 NmL min^{-1}.

2.12. Raman: Transient H_2+CO_2 vs He+CO_2

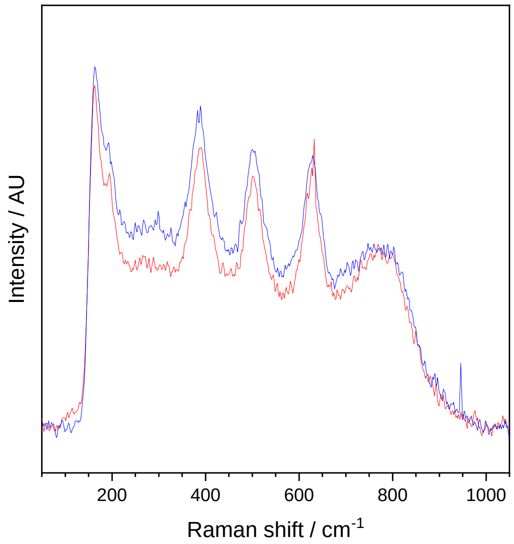

Figure S13 *In situ* characterization of 3 wt% Re/TiO_2 catalyst during transient H_2+CO_2 vs He+CO_2 experiment before (blue) and after (red) quasi steady state. Reaction conditions: ca. 10 mg catalyst, H_2/CO_2 = 3, T = 150 °C, P = 10 bar, F_{total} = 10 NmL min^{-1}.

2.13. DRIFTS: Transient H_2+CO_2 vs He+CO_2

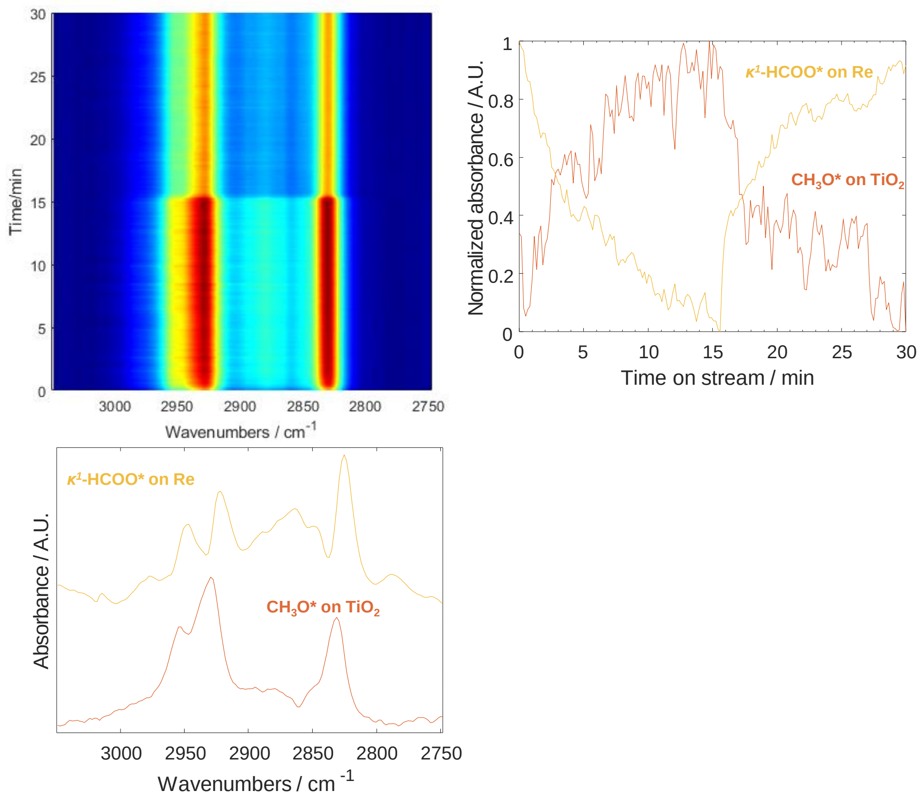

Figure S14 Transient DRIFTS study on CO_2 hydrogenation over 3 wt % Re/TiO_2 catalyst, time-resolved DRIFT spectra upon transient con-centration perturbation using H_2+CO_2 (0-15 mins) and H_2+He (15-30 min). Reaction conditions: 10 mg catalyst, H_2/He = He/CO_2 = 3, T = 150 °C, P = 10 bar, F_{total} = 10 NmL min^{-1}.

2.14. DRIFTS: Transient H_2+He vs He+CO_2

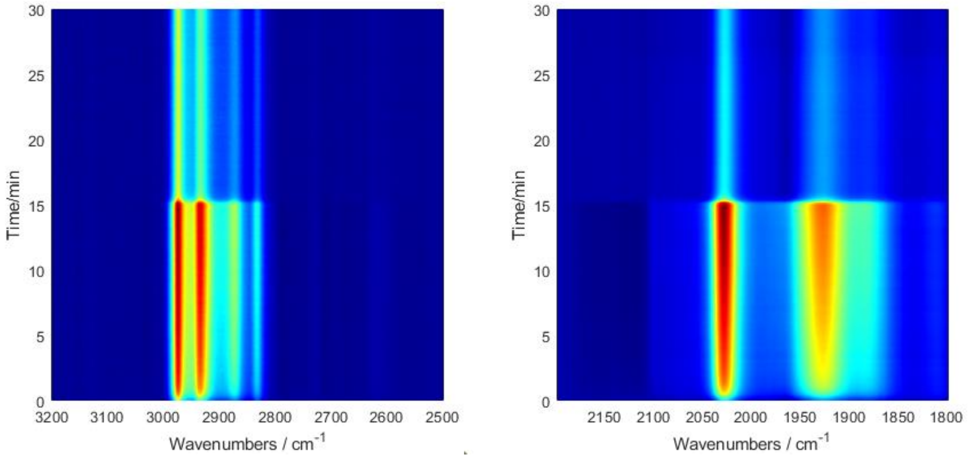

Figure S15 Transient DRIFTS study on CO_2 hydrogenation over 3 wt % Re/TiO_2 catalyst, time-resolved DRIFT spectra upon transient concentration perturbation using He+CO_2 (0-15 mins) and H_2+He (15-30 min). Components spectra obtained by MCR applied on the time-resolved DRIFT spectra (bottom-left). Concentration profiles of the spectra of the corresponding components obtained by MCR (top-right). Reaction conditions: 10 mg catalyst, H_2/He = He/CO_2 = 3, T = 150 °C, P = 10 bar, F_{total} = 10 NmL min^{-1}.

References

1 Bansode, A. & Urakawa, A. Towards full one-pass conversion of carbon dioxide to methanol and methanol-derived products. *Journal of Catalysis* **309**, 66-70 (2014). https://doi.org:https://doi.org/10.1016/j.jcat.2013.09.005

2 Urakawa, A., Bürgi, T. & Baiker, A. Sensitivity enhancement and dynamic behavior analysis by modulation excitation spectroscopy: Principle and application in heterogeneous catalysis. *Chemical Engineering Science* **63**, 4902-4909 (2008). https://doi.org:https://doi.org/10.1016/j.ces.2007.06.009

3 Jaumot, J., de Juan, A. & Tauler, R. MCR-ALS GUI 2.0: New features and applications. *Chemometrics and Intelligent Laboratory Systems* **140**, 1-12 (2015). https://doi.org:https://doi.org/10.1016/j.chemolab.2014.10.003

4 de Juan, A., Jaumot, J. & Tauler, R. Multivariate Curve Resolution (MCR). Solving the mixture analysis problem. *Analytical Methods* **6**, 4964-4976 (2014). https://doi.org:10.1039/C4AY00571F

5 Jackson, P. & Parfitt, G. D. Infra-red study of the surface properties of rutile. Deuterium exchange, carbon dioxide and but-1-ene adsorption. *Journal of the Chemical Society, Faraday Transactions 1: Physical Chemistry in Condensed Phases* **68**, 896-906 (1972). https://doi.org:10.1039/F19726800896

6 Uetsuka, H., Henderson, M. A., Sasahara, A. & Onishi, H. Formate Adsorption on the (111) Surface of Rutile TiO2. *The Journal of Physical Chemistry B* **108**, 13706-13710 (2004). https://doi.org:10.1021/jp049126z

7 Ting, K. W., Toyao, T., Siddiki, S. M. A. H. & Shimizu, K.-i. Low-Temperature Hydrogenation of CO2 to Methanol over Heterogeneous TiO2-Supported Re Catalysts. *ACS Catalysis* **9**, 3685-3693 (2019). https://doi.org:10.1021/acscatal.8b04821

8 Wang, Y., Wen, B., Dahal, A., Kimmel, G. A., Rousseau, R., Selloni, A., Petrik, N. G. & Dohnálek, Z. Binding of Formic Acid on Anatase TiO2(101). *The Journal of Physical Chemistry C* **124**, 20228-20239 (2020). https://doi.org:10.1021/acs.jpcc.0c06031

9 Wang, C.-y., Groenzin, H. & Shultz, M. J. Comparative Study of Acetic Acid, Methanol, and Water Adsorbed on Anatase TiO2 Probed by Sum Frequency Generation Spectroscopy. *Journal of the American Chemical Society* **127**, 9736-9744 (2005). https://doi.org:10.1021/ja051996m

10 Yang, D., Li, Y., Liu, X., Cao, Y., Gao, Y., Shen, Y. R. & Liu, W.-T. Facet-specific interaction between methanol and TiO2 probed by sum-frequency vibrational spectroscopy. *Proceedings of the National Academy of Sciences* **115**, E3888-E3894 (2018). https://doi.org:doi:10.1073/pnas.1802741115

11 Feracin, S., Buergi, T., Bakhmutov, V. I., Eremenko, I., Vorontsov, E. V., Vimenits, A. B. & Berke, H. Hydrogen/Hydrogen Exchange and Formation of Dihydrogen Derivatives of Rhenium Hydride Complexes in Acidic Solutions. *Organometallics* **13**, 4194-4202 (1994). https://doi.org:10.1021/om00023a024

12 Bolaño, S., Bravo, J., García-Fontán, S. & Castro, J. Rhenium pentahydride complexes: characterisation and protonation reactions. Crystal structure of ReH5L1L2 (L1=Ph2PO(CH2)2OPPh2; L2=P(OCH3)3, P(OCH2CH3)3). *Journal of Organometallic Chemistry* **667**, 103-111 (2003). https://doi.org:https://doi.org/10.1016/S0022-328X(02)02151-4

13 Cho, H.-G. & Andrews, L. Formation of HC:ReH3 in Methane Activation by Rhenium Atoms: Observation of the Elusive Methylidyne C−H Stretching Absorption. *Organometallics* **26**, 4098-4101 (2007). https://doi.org:10.1021/om070218v

14 Li, N., Xie, Y., King, R. B. & Schaefer III, H. F. Edge-Bridging and Face-Bridging Hydrogen Atoms in Trinuclear Rhenium Carbonyl Hydrides. *European Journal of Inorganic Chemistry* **2011**, 4626-4636 (2011). https://doi.org:https://doi.org/10.1002/ejic.201100476

15 Morris, R. H. Estimating the Wavenumber of Terminal Metal-Hydride Stretching Vibrations of Octahedral d6 Transition Metal Complexes. *Inorganic Chemistry* **57**, 13809-13821 (2018). https://doi.org:10.1021/acs.inorgchem.8b02314

16 Mino, L., Spoto, G. & Ferrari, A. M. CO2 Capture by TiO2 Anatase Surfaces: A Combined DFT and FTIR Study. *The Journal of Physical Chemistry C* **118**, 25016-25026 (2014). https://doi.org:10.1021/jp507443k
